# Assay development and screening of inhibitors targeting the SARS-CoV-2 2′-O-methyltransferase NSP16

**DOI:** 10.1016/j.pscia.2025.100076

**Published:** 2025-05-21

**Authors:** Mengtong Cao, Carl W. Trieshmann, Subodh Kumar Samrat, Hongmin Li, Yifei Wu, Steven P. Maher, Angela A. Bae, Zhong-Ru Xie, Robert J. Hogan, Y. George Zheng

**Affiliations:** a Department of Pharmaceutical and Biomedical Sciences, College of Pharmacy, University of Georgia, Athens, GA, United States; b Department of Pharmacology and Toxicology, R. Ken Coit College of Pharmacy, The University of Arizona, Tucson, AZ, 85721-0207, United States; c The BIO5 Institute, The University of Arizona, Tucson, AZ, 85721, United States; d Biological Chemistry Program, Department of Chemistry and Biochemistry, College of Science & College of Medicine, The University of Arizona, Tucson, AZ, 85721, United States; e Department of Molecular & Cellular Biology, College of Science, The University of Arizona, Tucson, AZ, 85721, United States; f College of Engineering, University of Georgia, Athens, GA, United States; g Center for Tropical and Emerging Global Diseases, University of Georgia, Athens, GA, United States; h Department of Biomedical Sciences, Animal Health Research Center, Department of Infectious Diseases, University of Georgia, Athens, GA, United States

**Keywords:** COVID-19, SARS-CoV-2, NSP16, NSP10, Assay design, Inhibitor screening, Drug discovery

## Abstract

The coronavirus disease-2019 (COVID-19) pandemic, etiologically caused by severe acute respiratory syndrome coronavirus 2 (SARS-CoV-2), has profoundly impacted the global health. While vaccines have been developed, they have shown limited efficacy in treating patients already under infection or preventing infection with emerging SARS-CoV-2 variants. The nonstructural protein 16 (NSP16), with the assistance of the nonstructural protein 10 (NSP10), is responsible for forming the Cap-1 structure, which is critical for viral replication and immune evasion through the 5′-capping of viral mRNA. As a result, NSP16/NSP10 has emerged as a promising target for antiviral treatment of coronaviruses. In this study, we aimed to discover small molecule inhibitors of NSP16/NSP10 by leveraging recent structural insights and combined tools of virtual and experimental screenings. We designed a simple scintillation proximity assay to enable biochemical testing for NSP16/NSP10 enzymatic activity and applied it to screen inhibitors from candidate hit compounds that are derived from molecular docking-based virtual screenings. We identified potential hits that inhibit the NSP16 activity with cellular efficacy. Together with structural analysis and chemotype categorization, this study lays the groundwork for novel antiviral therapeutics development against SARS-CoV-2 and related coronaviruses.

**Table:** Abbreviation list

Abbreviation	Definition
COVID-19	Coronavirus disease-2019
SARS-CoV-2	Severe acute respiratory syndrome coronavirus 2
NSP16	Nonstructural protein 16
NSP10	Nonstructural protein 10
ORFs	Open reading frames
pp1a	polyprotein 1a
pp1ab	polyprotein 1 ​ab
RTCs	Replicase-transcriptase complexes
PL^pro^	Papain-like protease
M^pro^	Main protease
SPA	Scintillation proximity assay
SAR	Structure-activity-relationship
IPTG	Isopropyl β-D-thiogalactoside
MTase	Methyltransferase
BSA	Bovine serum albumin
SAM	S-adenosylmethionine
SAH	S-adenosylhomocysteine
HTVS	High-Throughput Virtual Screening
QSAR	Quantitative structure activity relationship
MLR	Multiple linear regression
kNN	k-nearest neighbor
PLS	Partial Least Squares
RF	Random forest
HQSAR	Hologram quantitative structure activity relationship
MD	Molecular dynamics
PDB	Protein Data Bank
CPE	Cytopathic effect
EC_50_	Half-maximal effective concentration
CC_50_	Half-maximal cytotoxic concentration

## 1 Introduction

The outbreak of severe acute respiratory syndrome coronavirus 2 (SARS-CoV-2), which causes the coronavirus disease-2019 (COVID-19) pandemic, has significantly impacted the public health globally [1]. In response, the rapid development of antiviral agents such as antiviral drugs and vaccines hold promise to decrease and treat the occurrence of the coronavirus infection [2,3]. However, with the ongoing need for broad-spectrum antiviral drugs to treat SARS-CoV-2 infection and as SARS-CoV-2 continues to spread and evolve into various variants, such as Delta and Omicron, the demand for effective antiviral therapeutics is significantly increasing [4]. Thus, there remains an urgent need to discover or design new structural chemotypes to combat these variants. Coronaviruses are large, enveloped RNA viruses characterized by a single-stranded, positive-sense RNA genome [5,6]. They cause significant infectious diseases in humans and animals and have the largest known RNA viral genomes (∼30 ​kb), featuring essential molecular structures like a 5′ cap and 3′ poly (A) tail. Their genome's 3′ end contains sub-genomic RNAs encoding four key structural proteins: spike (S), envelope (E), membrane (M), and nucleocapsid (N) proteins [7]. This region also encodes nine accessory proteins that support viral replication. On the other hand, the 5′ end of the genome encodes two major open reading frames (ORFs), rep1a ORF and rep1b ORF, which are translated into two polyproteins, polyprotein 1a (pp1a) and polyprotein 1 ​ab (pp1ab) [8]. The pp1ab is produced through a ribosomal frameshifting from the rep1a reading frame into the rep1b ORF [[9], [10], [11]]. These polyproteins are subsequently cleaved by proteases into 16 non-structural proteins (NSP1-16) that form the replicase-transcriptase complexes (RTCs), which are essential for both viral replication and transcription [12]. The crucial non-structural proteins are NSP3, a papain-like protease (PL^pro^) [12]; NSP5, the main protease (M^pro^); the NSP7-NSP8 primase complex [13]; NSP12, an RNA-dependent RNA polymerase [14]; NSP13, which functions as an RNA helicase and triphosphatase [15,16]; NSP14, which has exoribonuclease and N7-methyltransferase activities [[17], [18], [19]]; NSP15, an endonuclease [20]; and NSP16, a 2′-O-methyltransferase [21].

An essential process in viral replication is the 5′-capping of mRNA, a conserved four-step process: (1) RNA 5′-triphosphatase removes the γ-phosphate from nascent RNA, forming ppNp-RNA [22]; (2) guanylyltransferase transfers GMP to form GpppNp-RNA; (3) NSP14 methylates guanine at the N7 position to produce Cap-0 (m7GpppNp-RNA) [23]; and (4) NSP16, activated by NSP10 [24], methylates the ribose 2′-OH of the first nucleotide, creating Cap-1 (m7GpppNm2′-Op-RNA) [25]. This Cap-1 structure is critical for viral replication and immune evasion, enhancing translation efficiency and preventing detection by host sensors such as IFIT and RIG-I [26,27]. Given its essential role and conservation across coronaviruses, the NSP16/NSP10 complex is a promising antiviral drug target, although effective inhibitors are yet to be developed [21].

**Fig. 1:**
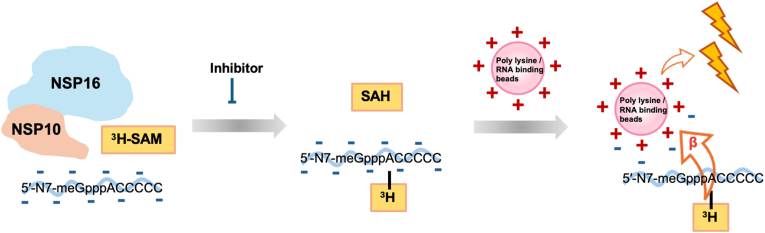
**Scheme of the RNA methyltransferase activity assay.** Measurement of NSP16/NSP10 methyltransferase activity using radiolabeled ^3^H-SAM. The ^3^H-methyl group was transferred to the capped RNA substrate and captured by different types of positively charged beads. The emitted beta particles stimulated light emission from the beads, which was quantified using a microplate scintillation counter to assess enzymatic activity.

To expedite drug discovery for RNA methyltransferases, in this study, we first developed a robust and sensitive biochemical assay for NSP16/NSP10 methyltransferase activity measurement and inhibitor screening (Fig. 1). This electrostatic interaction-based scintillation proximity assay (SPA) is sensitive, robust, and economically less expensive than the standard biotin-streptavidin binding based method. For NSP16/NSP10 inhibitor discovery, we conducted molecular docking-based virtual screenings to generate potential compound candidates for biological screening against NSP16/NSP10 activity. Biochemical and cellular assays were then used together to evaluate NSP16/NSP10 inhibitor potency and antiviral activity. The structure-activity-relationship (SAR) analysis was implemented to offer structural insights into the mode of inhibition and future analog design. Overall, our combined efforts of virtual screening, biochemical assays, and cellular tests yielded several NSP16/NSP10 inhibitors. Our findings provide a solid foundation for developing novel antiviral therapeutics against SARS-CoV-2 and related viruses.

## 2 Materials and methods

### 2.1 Expression and purification of NSP16/NSP10

The NSP16 gene sequence was codon optimized, synthesized, and inserted into the pET28a vector to generate the expression plasmid pET28a(+)-6His-NSP16 encoding full-length SARS-CoV-2 NSP16 by GeneUniversal. Similarly, the NSP10 gene sequence was codon optimized, synthesized, and inserted into the plasmid pGEX-6P-1 by GeneUniversal. The NSP16/NSP10 complex was obtained through co-expression of NSP16 and NSP10 plasmids. Briefly, *E coli* Rosetta (DE3) cells were co-transformed with the pET28(+)-6HisNSP16 and pGEX-6P-1-NSP10 plasmids on agar plate containing ampicillin and kanamycin. Next day, a transformed single colony was used to inoculate 50 ​mL LB media containing 10 ​μg/mL of ampicillin and 30 ​μg/mL of kanamycin. 50 ​mL culture were grown in a shaker with 250 ​rpm at 37 ​°C for overnight. A culture medium (1 ​L) was inoculated with the overnight grown pre-culture and further grown at 37 ​°C with 250 ​rpm until OD_600_ reached 0.6. Protein expression was induced by addition of Isopropyl β-D-thiogalactoside (IPTG) to a final concentration of 0.5 ​mM; and the cells was further grown at 16 ​°C for overnight. The cells were harvested by centrifugation at 7000 ​g for 10 ​min. The bacterial pellet was resuspended in a lysis buffer containing 20 ​mM HEPES, pH 8.5, 500 ​mM NaCl, 10% glycerol and 1 ​mM DTT. PMSF was added to the lysis buffer. The cells were lysed by sonication. The lysate was centrifuged at 20,000 ​g for 40 ​min. Cell lysate was loaded onto a glutathione Sepharose-4B column, pre-equilibrated with the cell lysis buffer. Upon extensive washing to remove non-specific binders with the lysis buffer, home-made 3C protease was added to the column for on-column digestion at 4 ​°C overnight to remove the GST-tag. Flow-through and two-column washing fractions was collected, combined, and concentrated before further purification using size exclusion chromatography with a Superdex 200 column equilibrated with 20 ​mM HEPES, pH 8.5, 150 ​mM NaCl, 10% glycerol and 1 ​mM DTT on an AKTA Pure 25M system. The fractions containing purified NSP16/NSP10 complex were pooled, concentrated, aliquoted, flash-frozen in liquid nitrogen and stored at −80 ​°C until further use.

### 2.2 Development and optimization of the NSP16/NSP10 biochemical assay

The initial methyltransferase (MTase) reactions were conducted in a reaction buffer consisting of 50 ​mM Tris-HCl (pH 7.5), 80 ​mM KCl, 5 ​mM DTT, 1.5 ​mM MgCl2, 0.01% Triton X-100, and 0.01% bovine serum albumin (BSA). The reaction mixtures (30 ​μL) were prepared containing 2 ​μM S-adenosylmethionine (SAM), which included 0.5 ​μM [^3^H]-SAM (Revvity, NET155V250UC) and 1.5 ​μM cold SAM, 1 ​μM substrate (5′-N7-meGpppACCCCC-biotin, Bio-Synthesis Inc.), and 0.25 ​μM NSP16/NSP10 complex. Reactions were incubated for 1 ​h and subsequently quenched by adding 30 ​μL of 100% isopropanol. The reaction products were transferred to a White 96-well Microplate with a Clear Bottom (Revvity, 6005040), and all reactions were performed in duplicate to ensure consistency. To determine the optimal quenching buffer, reactions were quenched with either 30 ​μL of 7.5M guanidine hydrochloride or 100% isopropanol. Additionally, to optimize the reaction temperature, the assays were performed at room temperature (23 ​°C) and 37 ​°C. All subsequent experiments were conducted at room temperature (23 ​°C) and quenched using 100% isopropanol for optimal performance.

To test the inhibitory effect of the reference compound S-adenosylhomocysteine (SAH) against NSP16/NSP10, reaction mixtures of 30 ​μL were prepared, each containing 2 ​μM S-adenosylmethionine (SAM), consisting of 0.5 ​μM radiolabeled [^3^H]-SAM and 1.5 ​μM unlabeled cold SAM, along with 1 ​μM substrate (5′-N7-meGpppACCCCC-biotin). Various concentrations of SAH were added to the reactions, with final concentrations of 0, 0.13, 0.26, 0.52, 1.04, 2.08, 4.16, and 8.33 ​μM. The reactions were initiated by adding 0.25 ​μM of the NSP16/NSP10 complex to the mixture. After 1 ​h of incubation, the reactions were quenched by the addition of 30 ​μL of 100% isopropanol. The reaction products were then transferred to a White 96-well Microplate with a Clear Bottom. 10 ​μL of 20 ​mg/mL of poly-lysine beads (Revvity, RPNQ0010) were added into each well. All reactions were performed in duplicate to ensure reproducibility. IC_50_ values were calculated from dose response curve using the Hill equation.

### 2.3 Biochemical screening of NSP16/NSP10 small molecule inhibitors and measurement IC_50_ of top hits

Reaction mixtures of 30 ​μL were prepared, containing 2 ​μM SAM (0.5 ​μM radiolabeled [^3^H]-SAM and 1.5 ​μM unlabeled cold SAM), along with 1 ​μM substrate (5′-N7-meGpppACCCCC-biotin). Selected compounds at final concentrations of 10 ​μM, 100 ​μM or 200 ​μM were added to the reaction mixture prior to the initiation of the enzymatic activity. The reactions were started by introducing 0.25 ​μM of the NSP16/NSP10 complex into the mixture. After the reaction was completed, it was quenched with 100% isopropyl alcohol. To further validate the inhibitory potential of the selected compounds, their IC_50_ values were determined using individual biochemical assays. For these assays, the incubation system is the same as previous described and the final concentrations of each compound were prepared through serial dilution, ranging from 1 ​mM to 1 ​μM. IC_50_ values were calculated from dose response curve using the Hill equation.

### 2.4 Anti-SARS-CoV-2 assay of NSP16 inhibitors in cultured cells

The Vero-E6 cell line (from African green monkey [Chlorocebus sp.] kidney epithelium), were obtained from ATCC (ATCC-CRL-1586) [28] and cultured in T175 flasks (Nunc,. 159910) in DMEM (Corning, 10-013-CV) with 10% FBS (Gibco, 16000-044) and 1x penicillin-streptomycin-neomycin solution (Fisher Scientific, 15640-055) in 5% CO_2_ at 37 ​°C. Cells were subpassaged at 90% confluence with Trypsin LE Express (Gibco, 12605-028) and maintained for less than 20 passages before thawing a new stock vial to maintain expression of ACE2. The day before infecting with SARS-CoV-2, 384-well assay plates (Grenier, 781090) were seeded with 4000 live cells in 40 ​μL media/well using a Biomek NXp (Beckman Coulter). Test compounds were supplied at 10 or 50 ​mM in DMSO and directly serially diluted (1:2, 12 points) in DMSO in a 384-well source plate (Grenier, 784261) using a Biomek 4000 (Beckman Coulter). For controls, 50 ​mM puromycin (Sigma, P8833, batch 92686), 50 ​mM molnupiravir (Caymen Chem, 29586, batch 0588683-4), 50 ​mM remdesivir (Caymem chem, 30354, batch 0588947-10), and 12 units/mL interferon beta (R&D Systems, 8499-IF/CF, lot DEQD0719071) were similarly added and diluted in each source plate. Two assay plates were started for each source plate, one for infection with SARS-CoV-2, and one for an uninfected cell cytotoxicity assessment. The following morning, all media was removed from assay plate wells and replaced with either 20 ​μL DMEM and 1% FBS (for plates to-be-infected) or 40 ​μL DMEM and 1% FBS (for uninfected cytotoxicity plates) using a Biomek NXp. Assay plates then treated by transferring 40 ​nL of compounds from the source plates using a pin tool affixed to an NXp. Assay plates were allowed to incubate for 3 ​h before plates designated for infection were moved into a Biosafety Level 3 laboratory. Assay plates were then infected with 20 μL/well SARS-CoV-2 WA1 (deposited by the Centers for Disease Control and Prevention and obtained through BEI Resources, NIAID, NIH: SARS-Related Coronavirus 2, Isolate USA-WA1/2020, NR-52281) diluted to 0.25 ​PFU/μL in DMEM with 1% FBS. Both the infected and cytotoxicity copies of each assay plate were then incubated for 72 ​h before endpoints. For infected plates, wells were fill with 20 ​μL Prestoblue Cell Viability Reagent (Fisher Scientific, A13261) diluted to 4.1x in PBS (leading to a final dilution of 1x in wells) and incubated at RT for 30min before reading fluorescence at 560 ​nm on a Synergy plate reader (Biotek). Cytotoxicity assay plates were emptied and fixed with 20 ​μL 4% paraformaldehyde and 10 ​μg/mL Hoechst 33342 (Fisher Scientific, H21492) in 1x PBS before imaging with a 4x objective on an ImageXpress Micro Confocal high content imager (Molecular Devices). Vero cell nuclei were counted using MetaXpress (Molecular Devices). Prestoblue viability data from infected plates and high content imaging data from uninfected cytotoxicity plates were loaded into CDD Vault for analysis and curve fitting. Cytopathic effect (CPE) in infected plates was normalized to remdesivir and DMSO controls; nuclei count from cytotoxicity plates were normalized to puromycin and DMSO controls. Selectivity was determined by dividing the EC_50_ of CPE by the CC_50_ from the cytotoxicity assessment. Potency and cytotoxicity were calculated from three independent experiments.

### 2.5 Virtual screening and top hits selection

*In-silico* docking was performed in Schrödinger Maestro software version 2022-23 installed and run remotely on the LINUX-based Georgia Advanced Computing Resource Center (GACRC) high-performance cluster and scored using Glide [29]. Initial screening was performed using crystal structure data obtained from Protein Data Bank (PDB) using ascension codes 6W4H ‘*1.80 ​Å Resolution Crystal Structure of NSP16/NSP10 Complex from SARS-CoV-2*’, 6WVN ‘*Crystal Structure of NSP16/NSP10 from SARS-CoV-2 in Complex with 7-methyl-GpppA and S-Adenosylmethionine*’ and 7R1T ‘*Crystal structure of SARS-CoV-2 NSP16/NSP10 in complex with the SS148 inhibitor’* [30,31]. Compounds were then selected for biological screening based upon results from High-Throughput Virtual Screening (HTVS), followed by Glide standard precision (SP) docking, MM/GBSA scoring and structural uniqueness to ensure a high diversity experimental set [32].

In addition to using a molecular modelling approach, we conducted a thorough literature review to identify reported and predicted inhibitors for inclusion in drug screening. These compounds were then compiled, cross-referenced to CDD Vault compounds, plated, and assigned lab identifiers for inhibitor-screening biological testing via scintillation proximity assay. In cases where structurally similar compounds were overly represented, a deliberate choice was made in screening to not pick large numbers of compounds within these high similarity clusters. Instead, a representative group of these compounds was taken forward to allow for some preliminary structure activity relationship while still maintaining high diversity test set. This research design allowed for the testing of compounds with a wide array of predicted interactions, chemical fingerprints and geometry within binding pocket.

### 2.6 Molecular modelling

Primary molecular modelling was performed using Schrödinger Maestro to assess predicted ligand conformation and interactions on compounds of interest and subsets of compounds to gain insight into structure activity relationships within the protein ligand complex as they relate to observed relative activities. The docking poses of screened active compounds with the lowest energies were examined to generate a working hypothesis, classify compounds and build a 3D pharmacophore model [29]. Secondary methods such as re-scoring docked complexes with a per-residue scoring in Maestro and tertiary modelling in Cresset's Flare program were also performed to inform the proposed model of inhibitory action and demonstrate the proposed conformations are consistent across modelling platforms using different force field and scoring functions [[33], [34], [35]].

To perform informatic analysis and quantitative structure activity relationship (QSAR) modelling, the resultant scintillation proximity assay (SPA) data obtained at 10 ​μM, 100 ​μM and 200 ​μM was imbedded with each compounds structural data. Data preparation was performed in Microsoft Excel and generated. CSV files containing compound identifier, compound structure and relative activity at experimental condition. Each experimental condition (10 ​μM, 100 ​μM, 200 ​μM) was treated as an *independent* data set for model building. In cases where there were experimental replicates with the same experimental conditions, the arithmetic average value was used. Ligand structures (SMILES) and chemical descriptors were calculated in Data Warrior & Chem Master respectively [36,37]. These descriptors served as the independent (x) variables to the biological response (y) variable during QSAR modelling and model training for machine learning (ML). QSAR model generation was performed manually and in an automated workflow in Chem Master using (1) Data Warrior descriptors (2) Chem Master descriptors and (3) Consensus descriptors. QSAR models were built using multiple linear regression (MLR), Partial Least Squares (PLS) regression, k-nearest neighbor (kNN), support vector regression (SVR) and random forest (RF) methods [[38], [39], [40], [41], [42]]. For manual QSAR model building data was split into 75/25 training/test sets using the Kennard-Stone Algorithm [43]. Hologram QSAR (HQSAR) was performed on each data set to observe the atomic contributions to observed potency within compounds of interest as calculated in our model.

Molecular dynamics (MD) simulations were performed on key molecules of interest and compared to endogenous ligand SAM and SAH. All MD simulations were performed using Desmond Maestro under academic licensing from D.E. Shaw Research (DESRES) [44]. Simulations were performed using identical system preparatory method and simulation conditions with the following exception: SAM and SAH poses were obtained from the available crystal structures while experimental ligand poses were obtained through molecular docking. In the simulations of inhibitors obtained from virtual screening, the poses with greatest energetic favorability as scored using Glide Extra-Precision (XP) docking were used. In all cases, protein-ligand complexes were prepared under the same force field (OPLS4), solvated in the same water model (TIP3P), energy minimized and simulated under identical NPT conditions for 500 ns [45,46]. Post MD simulation interaction analysis was performed and exported using the same method within Desmond's Simulation Interaction Diagram.

## 3 Results

### 3.1 Development and optimization of a biochemical assay for NSP16 activity measurement

We first undertook an assay development experiment aimed at generating a scintillation proximity assay (SPA) for quantitative detection of RNA methyltransferase activity with enhanced cost-effectiveness and robustness. Previously Khalili Yazdi et al. [47] developed a SPA method to screen inhibitors of the NSP16/NSP10 complex by utilizing the 5′-N7-meGpppACCCCC-biotin substrate captured on streptavidin PLUS FlashPlate. We explored the possibility of implementing the SPA strategy by utilizing simple electrostatic interaction between the substrate and SPA bead. Toward this goal, we compared three commercially available scintillant-encapsulating microspheric polymer particles: streptavidin-coated beads, RNA-binding beads, and poly-L-lysine beads. The use of streptavidin-coated beads relies on the high-affinity and specific binding of streptavidin to the biotin moiety of the RNA substrate, while RNA-binding beads and poly-lysine beads bind to the RNA substrate through electrostatic interactions between the positively charged beads and the negatively charged phosphate backbone of RNA molecules (Fig. 1). The negatively charged substrate, 5′-N7-meGpppACCCCC, was incubated with the NSP16/NSP10 enzyme and the radiolabeled methyl donor, ^3^H-SAM. During the reaction, NSP16/NSP10 transfers the ^3^H-labeled methyl group to the substrate. The enzymatic reactions were quenched with isopropyl alcohol (50 ​% final concentration) prior to being mixed with the SPA beads.

**Fig. 2:**
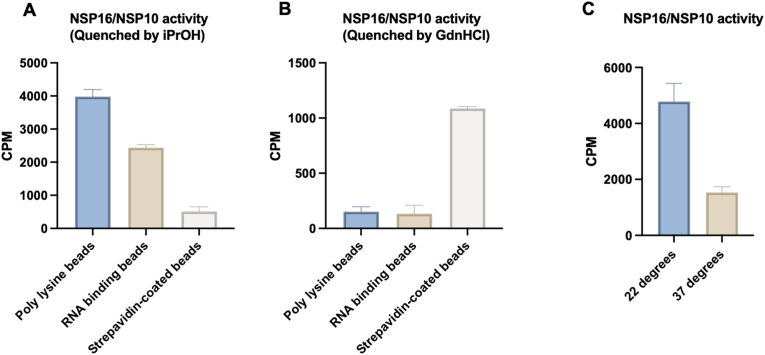
**Optimization of NSP16/NSP10 RNA methyltransferase assay beads and temperature.** (A) Comparison of three bead types for binding efficiency following MTase reactions quenched with isopropyl alcohol. (B) Binding efficiency of three bead types after MTase reactions quenched with guanidine hydrochloride and transferred to a white 96-well microplate. (C) Effect of incubation temperature (room temperature vs. 37 ​°C) on bead binding efficiency. Reactions were quenched with isopropyl alcohol and transferred to a white 96-well microplate.

As shown in Fig. 2A, among the three selected beads, poly-L-lysine beads emerged as the optimal choice, delivering the strongest signals in detecting NSP16/NSP10 methyltransferase activity, thereby offering a substantial improvement over the commonly used streptavidin-coated bead approach. Recognizing the importance of the quenching solution composition in the SPA test, we compared the performance of 7.5 ​M guanidine hydrochloride and 100% isopropanol as the quenching solution. Our results revealed different preferences based on bead type: streptavidin-coated beads performed optimally with guanidine hydrochloride as the quenching buffer, whereas poly-L-lysine beads and RNA-binding beads produced significantly better results when quenched with 100% isopropanol (Fig. 2B). Our results showed that, while streptavidin-coated beads performed optimally with guanidine hydrochloride as the quenching buffer, poly-lysine beads and RNA-binding beads produced significantly better results when quenched with 100% isopropanol, underscoring the importance of matching bead type with the appropriate quenching buffer to maximize assay efficiency. The differences in quenching buffer performance might arise from their distinct physicochemical properties. Isopropanol is a neutral organic molecule that denatures proteins while preserving nucleic acid‒poly-lysine electrostatic interactions, making it suitable for poly-lysine and RNA-binding beads. In contrast, guanidinium chloride, a charged molecule, not only denatured protein structures but also disrupted the electrostatic interactions critical for nucleic acid-poly-lysine binding. Therefore, guanidinium chloride is more suited for streptavidin-coated beads, but not for poly-L-lysine beads and RNA-binding beads.

**Fig. 3:**
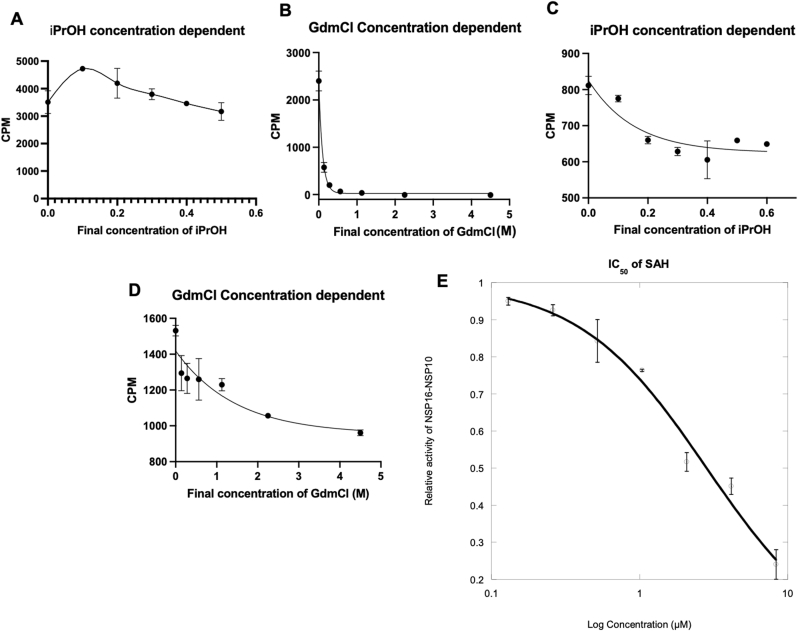
**Optimization of NSP16/NSP10 RNA methyltransferase assay quenching buffer and assay validation.** (A) Effect of varying concentrations of isopropyl alcohol on signal intensity using poly-lysine beads in the RNA methyltransferase assay. (B) Effect of varying concentrations of guanidine hydrochloride (GdnHCl) on signal intensity using poly-lysine beads. (C) Evaluation of isopropyl alcohol concentrations using streptavidin-coated beads in the RNA methyltransferase assay. (D) Evaluation of GdnHCl concentrations using streptavidin-coated beads. (E) Inhibition of NSP16/NSP10 activity by SAH using the optimized RNA methyltransferase assay.

Furthermore, we evaluated the influence of incubation temperature, comparing the assay outcomes at room temperature (22 ​°C) with that at 37 ​°C. The assay exhibited superior performance at room temperature rather than at 37 ​°C, suggesting that lower temperatures are more conducive to preserving the structural integrity of the NSP16/NSP10 complex and maximizing its enzymatic activity (Fig. 2C). To further investigate how the concentration of quenching buffer influences assay results, we conducted a concentration-dependent analysis using poly-lysine beads and streptavidin beads. For poly-lysine beads, quenching with isopropanol yielded consistent results across all concentrations tested (Fig. 3A). However, when guanidine hydrochloride was used as the quenching buffer, the signal intensity rapidly decreased as the concentration increased (Fig. 3B), which likely was due to guanidinium-caused disruption of the electrostatic interaction between the RNA substrate and the polymer beads as above mentioned. In contrast, for streptavidin beads when isopropanol was used as quenching buffer, the SPA signal intensities were poor at all the tested concentrations (Fig. 3C), consistent with observations in Fig. 2A. We posit that isopropanol may disrupt biotin-streptavidin interaction due to its denaturing effect on the streptavidin protein. Guanidine hydrochloride proved to be a more effective quenching buffer for streptavidin beads, although signal intensity still declined with increasing final concentrations (Fig. 3D).

To check if our optimized SPA method can be useful for NSP16 inhibitor discovery, we assessed the inhibitory activity of S-adenosylhomocysteine (SAH) against the NSP16/NSP10 complex, as previous studies have demonstrated that SAH inhibits NSP16's methyltransferase activity by binding to a critical groove on its surface. The IC_50_ value of SAH, determined using our optimized assay, was 3.0 ​μM, which aligns closely with previously reported values [48] and confirms the accuracy of our assay in measuring inhibitor potency (Fig. 3E). Collectively, these optimizations—spanning substrate capture, quenching conditions, and temperature—provide a refined and highly sensitive platform for screening inhibitors of the NSP16/NSP10 complex. This optimized assay offers a robust and reproducible method for the rapid identification of inhibitors of the NSP16/NSP10 complex and is of high value to the antiviral research community targeting coronavirus methyltransferase activity more broadly.

### 3.2 Molecular docking-based virtual screening to identify NSP16 inhibitors

**Fig. 4:**
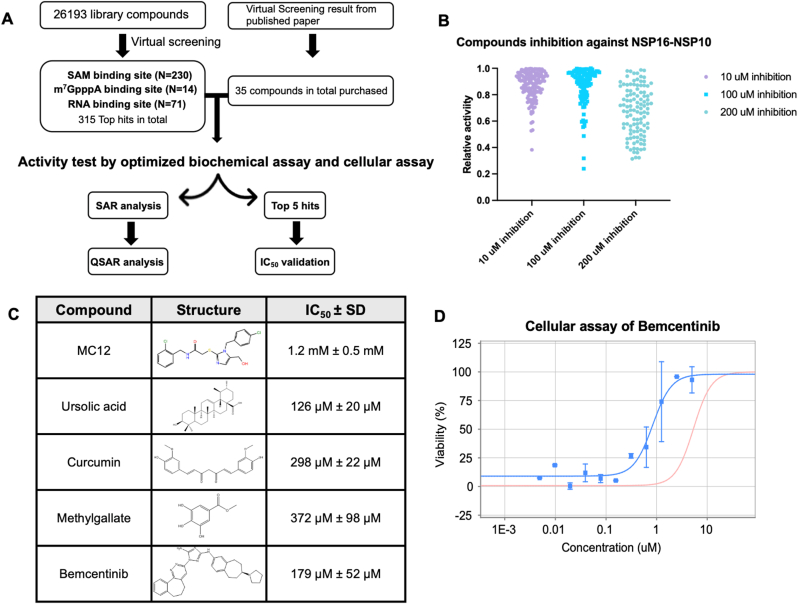
**Virtual screening scheme and biochemical assay top hits screening.** (A) Workflow of compounds collection, virtual screening and assays. (B) Reaction mixtures were prepared with SAM and 5′-N7-meGpppACCCCC-biotin as the substrate. Compounds were added to the reactions at final concentrations of 10 ​μM or 100 ​μM. Reactions were initiated by introducing 0.25 ​μM NSP16/NSP10 complex and quenched using 100 ​% isopropanol. The results highlight the impact of compound concentration on MTase activity. (C) Top hits from the biochemical assay. Compound structure and IC_50_ are shown. (D) Cellular assay result of bemcentinib against NSP16/NSP10.

To identify potential lead compounds targeting the NSP16/NSP10 complex activity, we conducted a molecular docking-based virtual screening across three distinct compound libraries from Life Chemicals, Inc.: natural product-like compounds library (913 compounds), and two pre-plated sets comprising 5120 and 20,160 compounds, respectively. The total number of screened structures was 26,193. Consensus molecular docking was performed using Schrödinger Maestro software. The crystal structure data sourced from the Protein Data Bank (PDB) using accession codes 6W4H, 6WVN, and 7R1T were adopted, representing key structural configurations of the NSP16/NSP10 complex, including a SS148 inhibitor-bound form [30,31]. Our docking-based screening targeted three critical binding sites within the complex—the SAM-binding site, the m7GpppA-binding site, and the RNA-binding site. This rational targeting strategy is based upon the fundamental mechanisms of NSP16/NSP10 methyltransferase activity and substrate recognition. The SAM-binding pocket is the catalytic site of methyl transfer, the m7GpppA site is essential for alignment of the RNA cap for modification, and the RNA-binding site is necessary for tethering the enzyme to RNA and ensuring specificity. This design was critical in allowing the targeting of co-factor and substrate recognition which proved essential in later development of proposed hypothesis of inhibitor action and classification. Following molecular docking, compounds were ranked by predicted binding energies and top binders were selected into a non-redundant list. The top 315 compound hits were extracted from the physical compound stocks for biochemical tests based on the molecular docking results. In addition, we purchased 35 compounds from commercial vendors that were previously proposed as potential inhibitors based on virtual screenings from the literature [[49], [50], [51], [52], [53], [54], [55], [56], [57], [58]]. As such, a total of 350 compounds were obtained and subjected to the next biochemical test. (Fig. 4A). Of note, we used RDKit and DataWarrior to screen for motifs of pan-assay interference compounds (PAINS) among the selected 350 compounds, and only a few purchased compounds, such as curcumin and methyl gallate, were identified as PAINS. We made a deliberate decision not to exclude these few compounds solely based on PAINS flags at this early stage of the screening process.

Docking results showed that the experimental ligands had comparable binding affinities to the endogenous co-factor SAM and inhibitor SAH, with predicted affinities ranging from −8.238 to −10.035 ​kcal/mol, though none surpassed these values. Despite this, many screened molecules displayed higher ligand efficiencies than SAM or SAH. The highest affinities were observed in small O- and N-heterocycles, with common scaffolds including quinoline, indole, furans, thiophenes, imidazole, isoxazole, pyridine, and pyrimidine. A frequent feature among top binders was a central amide flanked by aromatic heterocycles, particularly N-heterocycles, often extended with rings like pyridine, piperazine, or azetidine. The conformation of docked ligands was also considered relative to the endogenous ligand SAM which afforded clustering by pose. This clustering proved essential when interpreting results of biochemical assay and informing classification system for inhibitor development.

### 3.3 Biochemical test of the top virtual hits against NSP16/NSP10 using the optimized methyltransferase assay

The top 350 compounds identified through virtual screening were subjected to biochemical assays to evaluate their inhibitory potential. Initial compound selection and screening at 100 ​μM and 10 ​μM targeted SAM-binding, m7GpppA-binding and RNA-binding sites. During the initial screen of about 200 compounds, weak inhibitory effects were observed at 10 ​μM and 100 ​μM. Consequently, subsequent testing employed a higher concentration of 200 ​μM to better assess inhibitory activity. This two-tiered research design afforded the ability to perform downstream QSAR modelling while simultaneously testing and validating previous computational work predicting potential NSP16/NSP10 inhibitors.

Each compound was dissolved in 100% DMSO to prepare a 10 ​mM stock solution and further diluted by deionized water to the proper concentrations. In our assay, the compounds were tested at 10 ​μM and 100 ​μM or 200 ​μM, to determine their inhibitory effects (Fig. 4B). The reactions were prepared in an incubation mixture containing SAM and 5′-N7-meGpppACCCCC-biotin as substrate and they were diluted with the reaction buffer consisting of 50 ​mM Tris-HCl (pH 7.5), 80 ​mM KCl, 5 ​mM DTT, 1.5 ​mM MgCl_2_, 0.01% Triton X-100, and 0.01% BSA to reach the calculated concentration. 10 ​μM, 100 ​μM or 200 ​μM final concentration of compounds were added, and the reactions were initiated by introducing 0.25 ​μM of the NSP16/NSP10 complex to the mixture and quenched by 100% isopropyl alcohol. 10 ​μL of 20 ​mg/mL SPA beads were added and scintillation counting was conducted on Microbeta instrument. The biochemical assay results showed that at 10 ​μM, 100 ​μM or 200 ​μM final concentration, there are about 10 compounds that inhibited more than 30% of the relative activity of the NSP16/NSP10. We focused on analyzing compounds that could inhibit more than 30% of the enzymatic activity at 100 ​μM final concentration and have a lower inhibitory effect at 10 ​μM. There are 5 compounds in total that fit the requirement, 1 from our virtual screening and 4 from the virtual screening reported in the literature.

We further tested the IC_50_ of those 5 top hits against NSP16/NSP10 by performing individual biochemical assay. The final concentration of each compound ranged from 1 ​mM to 1 ​μM, prepared through serial dilution. Among the tested compounds, ursolic acid and bemcentinib showed IC_50_ values below 200 ​μM, suggesting their potential as effective inhibitor. Curcumin and methylgallate exhibited IC_50_ values of 298 ​μM and 372 ​μM, respectively. These four compounds were identified from the previous virtual screening studies [53,54,56]. MC12, the compound that got from our docking screening, had an IC_50_ of 1.2 ​mM (Fig. 4C). While its IC_50_ value of 1.2 ​mM was higher than those of the other candidates, it remains noteworthy in light of our computational predictions and may represent a different mechanism or interaction profile. Overall, this combination of biochemical screening and IC_50_ determination allowed us to refine our list of potential candidates and validate computational predictions, highlighting bemcentinib and ursolic acid as particularly promising inhibitors for further investigation.

### 3.4 Cellular test of the NSP16/NSP10 inhibitors for antiviral SARS-CoV-2 activity

We extended our investigation to cellular assays to evaluate the effects of the selected compounds under more physiologically relevant conditions. For these assays, Vero-E6 cells were seeded into 384-well plates and treated with serially diluted test compounds and appropriate controls prior to infection with SARS-CoV-2 WA1, which then caused cytopathic effect (CPE) in the Vero-E6 lawn. Each assay was started with a matching uninfected plate to assess cytotoxic effects. After 72 ​h of incubation, CPE and cytotoxicity were analyzed, and the selectivity of each compound was determined by comparing its EC_50_ (half-maximal effective concentration in the CPE assay) to its CC_50_ (half-maximal cytotoxic concentration) across three independent experiments. Interestingly, while MC12, ursolic acid, curcumin, and methylgallate demonstrated inhibitory activity in our biochemical assays, these compounds failed to exhibit significant antiviral effects in the cellular assays. This discrepancy highlights the importance of evaluating candidate compounds in cellular systems to account for factors such as cell permeability, metabolic stability, and off-target effects, which can influence efficacy (Supplementary Fig. S1). In contrast, bemcentinib emerged as a promising candidate, with an EC_50_ below 1 ​μM (Fig. 4D). This compound showed an EC_50_ of 0.583 ​μM and CC_50_ of 2.44 ​μM, which yielded a therapeutic window of 4.19. These results positioned bemcentinib as a strong contender for further development and NSP16/NSP10 is the possible target of it. The combination of its potent inhibition of the NSP16/NSP10 complex *in vitro* and its robust activity in cellular assays underscores its potential as a lead compound for therapeutic intervention against SARS-CoV-2.

### 3.5 Binding modality classification of the tested NSP16/NSP10 inhibitors

**Fig. 5:**
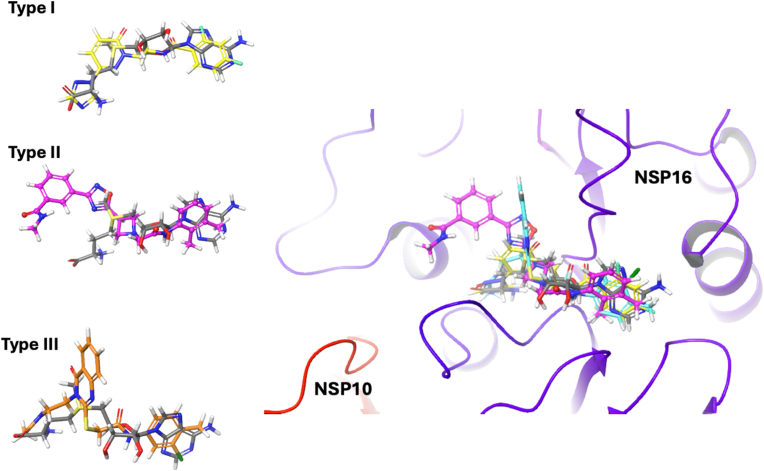
**Inhibitor classification using overlay of s-adenosylmethionine (SAM) to demonstrating binding.** Three binding modalities were observed which allowed classification of inhibitor types. Type I compounds occupy the same space in the NSP16 SAM binding site and overlay with the base, sugar and methionine of SAM. Type II compounds occupy the same regions as the base and sugar moiety of SAM but extend outwards blocking substrate recognition or disrupting NSP10-16 interface. Type III compounds overlay with the base, sugar and methionine of SAM but also have extended functionality like Type II compounds. Relative position of classified compounds shown within NSP16 (purple) and proximity to interacting regions of NSP16 and NSP10 (red).

Based on the biochemical activity data of the 350 top hits, we performed a series of computational studies in order to understand the possible mechanism(s) of inhibition, identify the structural features common to active compounds, and generate predictive pharmacophore model(s) to enrich future NSP16/NSP10 inhibitor design in the field. When overlaying docked ligands with the reference ligand SAH, some compounds aligned well with its base, sugar, and methionine components, while others showed alternative binding modes, either extending into the methionine binding pocket or instead blocking substrate recognition or interacting with residues critical for stabilizing the NSP16/NSP10 dimerization interface. To distinguish between these compounds, we classified the compounds which overlay with base, sugar and methionine of SAH as type I compounds, those which overlay with base, sugar and extended towards the hetero-dimerization interface as type II compounds, and those which overlay with the base, sugar, methionine *and* extended into the hetero-dimerization interface as type III compounds (Fig. 5). The key distinction between type II and type III compounds is that type II ligands are *not* predicted to extend into methionine binding pocket of SAM. Critically, both type II and type III compounds possess bulky functional groups which also likely interfere with substrate recognition.

Using this classification grouping, we examined the representation of these compounds and their biological activity. Type I compounds showed the least representation among those with 30% or higher relative inhibition, with none present in the most active compounds screened at 10 ​μM. Type II compounds were the most frequently observed across all activity levels, showing strong enrichment among compounds with higher inhibition. While Type III compounds showed moderate representation at higher concentrations (200 ​μM and 100 ​μM), but their presence decreased at lower concentrations. At the lowest screening concentration (10 ​μM), all type III compounds were absent among the most active compounds, with only a single exception. Taken together, this molecular modelling suggests that the most biologically active compounds in this model did *not* extend into the methionine binding region of the active site, but rather extended outwards to interact with key residues at NSP16/NSP10 interface or sterically block substrate recognition with their bulky sterically hindering functional groups.

### 3.6 Structure-activity relationship (SAR) analysis of NSP16/NSP10 complex

**Fig. 6:**
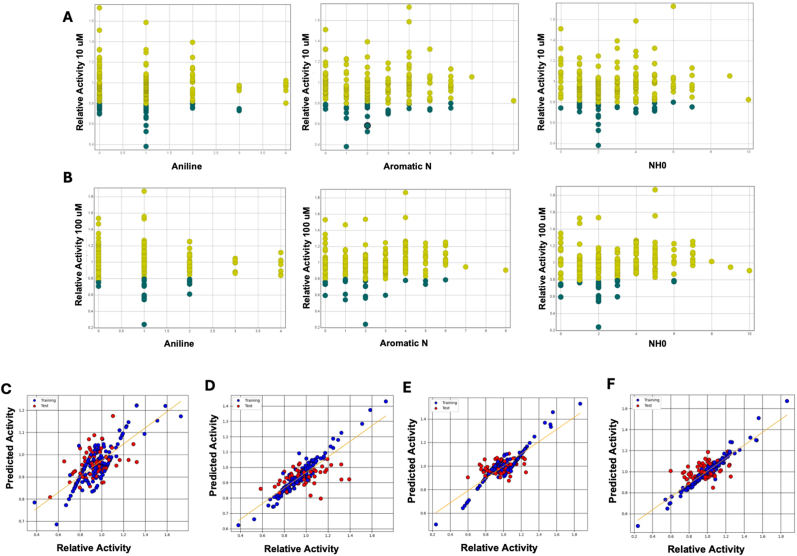
**Structure Activity Relationships and QSAR model development using Machine Learning.** (A and B) Illustrating consistently observed (A) trends in nitrogenous functional groups in 10 μM screening, and (B) trends in nitrogenous functional groups in 100 μM screening. (C–F) QSAR model development highlighting improvement using Random Forest (RF) over Support Vector Regression (SVR): (C) 10uM model generated using SVR; (D) 10uM model generated using RF; (E) 100uM model generated using SVR (F) 100uM model generated using RF.

To generate a quantitative SAR (QSAR) model, chemical descriptors were calculated and individually plotted against experimentally determined relative activity in Data Warrior. Despite the initial diversity of compounds, which made the SAR analysis challenging, some trends were noticeable. Active compounds showed a preference for nitrogen-containing structures, optimal H-bond donors/acceptors, and specific counts of benzene rings and heteroatoms. Amides, amines, aniline, and aromatic nitrogen appeared more frequently in active compounds and were generally associated with higher potency, even among inactive compounds (Fig. 6A and B). For amides, potency increased as the amide count rose, with 1–2 amides being typical (e.g., compounds with internal amide bonds), and compounds with three amides (like those with a lactam ring and internal amide bond) showing even greater activity. A similar trend was observed with aniline groups, where higher aniline counts were linked to activity, though the most potent compounds at 10 ​μM often had a single aniline group, marking a notable outlier. Another trend was the enrichment of high-potency compounds based on H-bond acceptor count, with all the most potent compounds in the 10 ​μM dataset possessing five H-bond acceptors (Supplementary Fig. S2).

Next, a series of QSAR models of increasing dimensionality were generated using Chem Master's internal utility to compute industry-standard RDKit molecular descriptors and Morgan fingerprints in an automated workflow to generate and test ∼5000 models. Using this Auto-QSAR method on the 10 ​μM, 100 ​μM and 200 ​μM data sets we generated reasonable models using support vector regression, random forest, k-nearest neighbor and support vector regression algorithms (Fig. 6C–F). Using the 10 ​μM data we generated an SVR model (SVR, model 3125) which had a low test set RMSE (0.132) and reasonable training set R^2^ (0.579) while a random forest model (RF, model 4454) yielded a slightly higher test set RMSE (0.143) and an improved R^2^ (0.841). These models are imperfect but represent a reasonable starting point for future NSP16 inhibitor design where there is a dearth of published inhibitor data; notably the models generated using 10 ​μM & 100 ​μM are consistent and with acceptable parameters given the diversity of compounds screened while the 200 ​μM models have poorer test set RMSE owing to the multiple proposed binding modalities used in this phase of compound selection (Supplementary Fig. S3A and B). In each data set, the algorithm which performed consistently well was random forest.

**Fig. 7:**
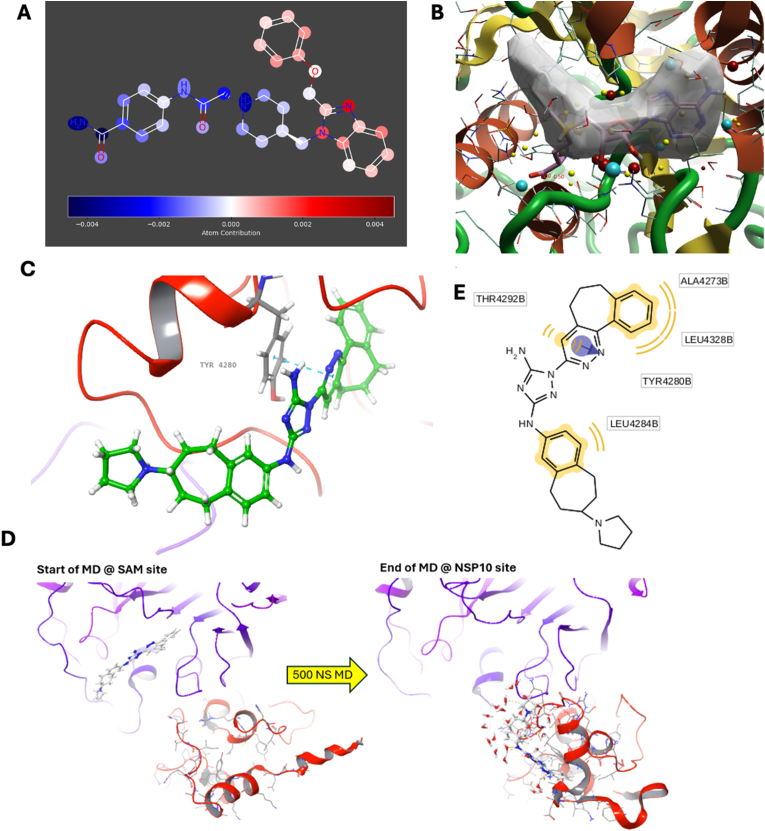
**2D, 3D QSAR modelling, molecular dynamics of bemcentinib and proposed binding of bemcentinib on NSP10.** (A) Heat map of Type II compound F5754-0421 showing atomic contributions to activity in a 2D-holographic QSAR model generated using the PLS method. Bulky aromatic groups are highlighted as key contributors. (B) 3D-QSAR model from Cresset's Activity Atlas displaying the average shape of potent inhibitors, overlapping with the base and sugar regions of SAM but extending beyond the methionine moiety. (C) Representative frame from a molecular dynamics simulation showing bemcentinib dissociating from the SAM binding site and interacting with NSP10, inducing conformational changes. (D) Predicted 3D binding pose of bemcentinib in NSP10, showing π–π interaction with Tyr4280 near the NSP10/NSP16 interface. (E) 2D pharmacophore model generated by LigandScout highlighting interaction with Tyr4280 and dominant hydrophobic features at the predicted binding site.

Using circular Morgan fingerprints, a 2D HQSAR model was generated with the partial least-squares (PLS) method in Chem Master to identify molecular fragments linked to potency, producing heatmaps for key compounds such as bemcentinib and representative Class I, II, and III inhibitors (Supplementary Fig. S3C–E). The model aligned with our inhibitor classification system, highlighting that features like the bulky ring system in Class II compound F5754-0421 likely disrupt substrate recognition and destabilize NSP16/NSP10 dimerization, correlating with potency (Fig. 7A). Per-residue energy calculations using Glide, informed by the crystallographic structures used for molecular docking, showed modest correlations between docking interactions at dimerization residues and activity (Supplementary Fig. S4) [31]. To ensure robustness, docking and QSAR modeling were replicated using Cresset's Flare and Activity Atlas, which confirmed consistent correlations between ligand structure, electrostatic and hydrophobic interactions, and potency across datasets (Supplementary Fig. S5). Activity Atlas further highlighted SAM-binding conformations of Type II compounds as most associated with potent NSP16 inhibition (Fig. 7B).

### 3.7 Molecular dynamic simulation

To investigate the dynamic behavior of bemcentinib, class I, II, and III compounds upon binding with the NSP16/NSP10 complex compared to the endogenous co-factor SAM and inhibitor SAH, we conducted molecular dynamics (MD) simulations using Desmond Maestro. Comparing the co-factor SAM to SAH showed clear differences in interacting residues and interaction type over the course of the MD simulation (Supplementary Fig. 6). SAM and SAH showed conserved interactions with Asp 6897 through water bridges and H-bonding with the two hydroxyl groups on their central sugar (see Fig. 10d supplemental). Distinctly, SAM formed ionic interactions with Asp 6928 and Lys 6968, whereas SAH engaged Asp 6928 through extensive H-bonding and water bridges, with no interaction observed with Lys 6968. Additionally, SAH's H-bonding at Asp 6912 and Cys 6913 was minimal. SAM consistently maintained water bridges and H-bonding at Tyr 6930, a feature much weaker with SAH.

Using these interactions as a reference, we conducted MD simulations on a series of active compounds and selected the most potent from each class (I, II, and III) as representative of each unique binding mode. For comparison, we selected F0464-0032 (class I, 0.7906 relative activity at 100 ​μM), F5754-0421 (class II, 0.2395), and F0913-3707 (class III, 0.5854). All compounds showed consistent interactions with Asp 6897 and Tyr 6930. Notably, as the strength and duration of interaction with Asp 6897 increased, potency also correlated, with the type II compound (F5754-0421) maintaining a stable ionic interaction. H-bonding with Asp 6897 was observed for all compounds, with diminishing frequency linked to lower potency. The type II and III compounds interacted with Lys 6968 similarly to SAM, primarily through hydrophobic, H-bonding, or water-bridging interactions. F5754-0421 also showed a transient ionic interaction with this residue. Type III compounds mirrored SAM/SAH interactions with the methionine region but with enhanced stability. In contrast, type I compounds briefly engaged with this region, highlighting its specificity for SAM. This pattern, along with the increased potency of type II and III compounds, aligns with bi-functional inhibition strategies in methyltransferase research.

The lead compound identified, bemcentinib, was analyzed separately due to its unique binding mode (Fig. 7C). Previous studies suggested bemcentinib would strongly bind at the NSP16/NSP10 interface, which our molecular modeling confirms [59]. Unlike typical SAM site binders, bemcentinib showed poor docking scores using Glide and quickly dissociated from this site in our MD simulation (Fig. 7D). However, it rapidly bound to NSP10, causing an observable conformational change in the enzyme. A second MD simulation confirmed stable binding near the NSP16/NSP10 interface, with π-π stacking at NSP10's Tyr 4280 and bemcentinib's pyridazine ring, along with H-bonding and water-mediated interactions. The three-ring structure of bemcentinib wedges between two NSP10 alpha helices. Overlaying the bemcentinib-bound NSP16/NSP10 complex with the SAM-bound version reveals conformational shifts, and protein-protein interaction analysis indicates bemcentinib reduces hydrogen bonding between the two proteins (Supplementary Fig. S7).

## 4 Discussion

The continuous evolution of SARS-CoV-2 variants and their substantial impact on global health underscores the critical need for effective antiviral therapeutics targeting essential viral infection pathways. In this work, we first developed and optimized a biochemical assay to screen inhibitors of the NSP16/NSP10 complex, a key enzyme involved in the viral replication cycle of coronaviruses. The NSP16/NSP10 complex mediates the methylation of the 5’ cap structure of viral mRNA, a modification that plays a pivotal role in immune evasion and viral propagation. By utilizing poly-lysine beads for SPA signal readout and isopropanol as a quenching buffer, the assay achieved significantly robust signal detection and sensitivity, establishing a reliable platform for identifying NSP16/NSP10 inhibitors. This method, based on the electrostatic interaction between negatively charged RNA and positively charged beads, can be applied broadly in high-throughput screening for inhibitors of various RNA methyltransferases and it is more cost-effective compared to other approaches like using SPA beads. In principle, this method could be implemented to measure DNA methyltransferase activity as well. Through molecular docking-based virtual screening, we identified several promising inhibitors that target critical binding sites within the NSP16/NSP10 complex.

Among these top hits, bemcentinib emerged as a lead compound with an EC_50_ below 1 ​μM in cellular assays, highlighting its potential to effectively inhibit NSP16/NSP10 activity and thereby disrupt viral replication. Bemcentinib's low EC_50_ and its specificity toward the NSP16/NSP10 complex make it a strong candidate for further preclinical development as a targeted antiviral agent. It will be important to conclusively resolve bemcentinib's mechanism of action in NSP/NSP10 inhibition. Previously, bemcentinib was identified as a small-molecule inhibitor of AXL kinase which may also target host cell pathways rather than the virus directly [60]. By inhibiting AXL, which facilitates viral entry and suppresses type I interferon responses, bemcentinib may enhance the host's immune response and limit the ability of SARS-CoV-2 to infect cells. This previous study and our current *in vitro* and cellular data support further investigation into chemical modification or drug repurposing of bemcentinib.

In addition to identifying bemcentinib as a lead compound for future development, we characterized many other small molecule natural products which may be further optimized or linked in a fragment-based drug design approach in the future. Ursolic acid, primarily extracted from medicinal plants such as *Mimusopscaffra*, *Ilex paraguarieni*, and *Glechoma hederacea*, exhibits potent anti-inflammatory, anti-bacterial, antioxidant, anti-cancer, and anti-diabetic properties [61]. Notably, it interacts with NSP15 and M proteins and has been shown to potentially block the M^pro^ enzyme, with a high docking score of −8.7 ​kcal/mol, suggesting strong binding and inhibitory effects [62]. In our study, we identified NSP16/NSP10 as a novel target for ursolic acid. Similarly, curcumin, another bioactive compound, has been reported to inhibit SARS-CoV-2 M^pro^ more effectively than several marketed drugs in silico, and it also blocks viral entry via the S protein and ACE2 receptor [63]. Our findings suggest that curcumin may also target the NSP16/NSP10 complex in SARS-CoV-2 treatment. Methylgallate, a phenolic compound known for its antioxidant and anti-inflammatory effects, has demonstrated antiviral activity against HIV, influenza, and dengue viruses [64], although no studies have yet explored its effects on SARS-CoV-2. Given its profile, methylgallate could serve as a new lead compound for inhibiting the NSP16/NSP10 complex in SARS-CoV-2.

Structural insights from our per-residue scoring, QSAR analysis and molecular dynamics simulations provided further insight into the likely binding interactions of active compounds with the NSP16/NSP10 complex. Under our working hypothesis, compounds classified as type II and III demonstrated greater inhibitory effects, likely by disrupting hydrophobic interactions between NSP10 and NSP16 at critical interfaces previously identified in the complex or interfering with substrate recognition [30]. These findings not only enhance our understanding of the functional dynamics of the NSP16/NSP10 complex but also point to new mechanistic strategies for designing inhibitors that specifically target these critical hydrophobic interactions and destabilize the complex.

## 5 Limitation

While this study provides a valuable foundation for the discovery of SARS-CoV-2 NSP16/NSP10 methyltransferase inhibitors, several limitations should be acknowledged: (1) the identified compounds show only modest biochemical potency (e.g., bemcentinib IC_50_ ​= ​179 ​μM; MC12 IC_50_ ​= ​1.2 ​mM), which may limit their potential for lead optimization; (2) variability in IC_50_ values, particularly for MC12, indicates a need for further assay replication and refinement; (3) the lack of direct confirmation of NSP16/NSP10 as the cellular target raises the possibility of off-target effects, including known AXL kinase inhibition by bemcentinib [65]; (4) the antiviral effects were assessed using qualitative cytopathic effect assays, and quantitative virological methods would strengthen these findings; and (5) the virtual screening focused on the NSP16 catalytic site without orthogonal validation, limiting confidence in the predicted binding interactions, though further pharmacophore refinement could improve predictive power.

## 6 Conclusion

In this study, we successfully developed and optimized a robust, sensitive screening platform for identifying RNA methyltransferase inhibitors targeting the NSP16/NSP10 complex. Several promising inhibitor candidates were identified, offering novel structural chemotypes for future medicinal chemistry development. Given the conserved and essential role of NSP16/NSP10 in coronavirus replication, these inhibitors hold significant promise as broad-spectrum antiviral therapeutics. Further medicinal chemistry studies should refine pharmacophore models to enhance specificity for NSP16/NSP10, optimize inhibitor potency, and reduce potential off-target effects. Importantly, targeting the NSP16/NSP10 interaction, rather than the highly conserved SAM-binding site, represents an innovative approach that may yield inhibitors with improved selectivity and safety profiles.

## CRediT authorship contribution statement

**Mengtong Cao:** Writing – review & editing, Writing – original draft, Investigation, Formal analysis, Data curation, Conceptualization. **Carl W. Trieshmann:** Writing – review & editing, Writing – original draft, Visualization, Methodology, Investigation, Data curation. **Subodh Kumar Samrat:** Writing – review & editing, Data curation. **Hongmin Li:** Writing – review & editing, Supervision, Investigation, Funding acquisition, Data curation. **Yifei Wu:** Writing – review & editing, Investigation, Data curation. **Steven P. Maher:** Writing – review & editing, Writing – original draft, Methodology, Investigation, Data curation. **Angela A. Bae:** Writing – review & editing, Writing – original draft, Investigation, Data curation. **Zhong-Ru Xie:** Writing – review & editing, Data curation. **Robert J. Hogan:** Writing – review & editing, Methodology, Funding acquisition. **Y. George Zheng:** Writing – review & editing, Supervision, Project administration, Methodology, Investigation, Funding acquisition, Conceptualization.

## Data availability

All data generated or analyzed during this study are included in this article and its supplementary information files.

## Ethics approval

Not applicable.

## Declaration of generative AI in scientific writing

Not applicable.

## Funding information

This study was supported by grants AI158176 to Y.G.Z. and AI175435 and AI177149 to H.L. from the 10.13039/100000060National Institute of Allergy and Infectious Diseases (NIAID), the 10.13039/100000002National Institutes of Health (NIH). Y.G.Z. was additionally supported by a 10.13039/100000057National Institute of General Medical Sciences grant GM149230. H.L. was also supported by the 10.13039/100000060NIH grants: AI161845 and AI131669.

## Conflict of interest statement

The authors declare that they have no financial or non-financial competing interests related to this work.
