# Supplementary material for: Assay development and screening of inhibitors targeting the SARS-CoV-2 2′-O-methyltransferase NSP16
Source: Pharm Sci Adv. 2025 May 21;3:100076. doi: 10.1016/j.pscia.2025.100076 (PMC12709967; doi:10.1016/j.pscia.2025.100076)
Supplement: Multimedia component 1 [file mmc1.pdf]

Supplementary Table 1. List of compounds as NSP16 inhibitor candidates based on the virtual screenings reported in the literature.

	Compound	Structure	Literature
1	**Isoquercetin**<br>[2-(3,4-dihydroxyphenyl)-5,7-dihydroxy-3-(((2*S*,3*R*,4*S*,5*S*,6*R*)-3,4,5-trihydroxy-6-(hydroxymethyl)tetrahydro-2*H*-pyran-2-yl)oxy)-4*H*-chromen-4-one]<br><br>CAS: 482-35-9	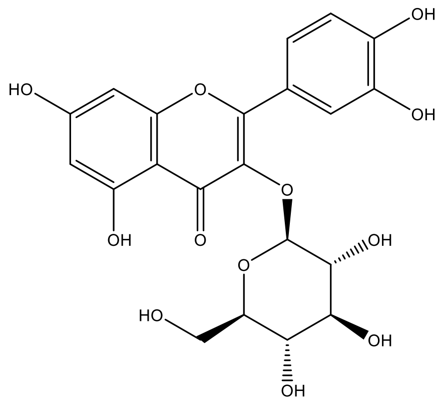	[1]
2	**Amentoflavone**<br>[8-(5-(5,7-dihydroxy-4-oxo-4*H*-chromen-2-yl)-2-hydroxyphenyl)-5,7-dihydroxy-2-(4-hydroxyphenyl)-4*H*-chromen-4-one]<br><br>CAS: 1617-53-4	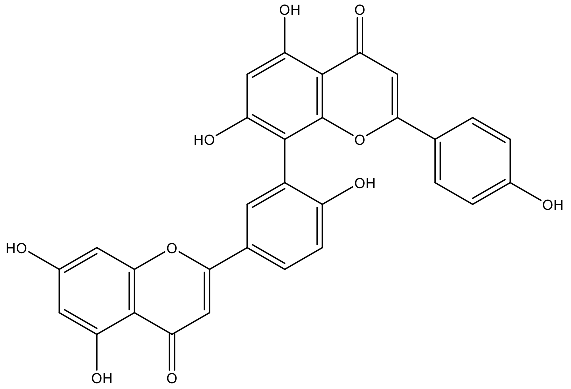	[2, 3]

3	**Cepharanthine**<br>[(1^5S,3^1R)-3^6,5^4-dimethoxy-1^6,3^2-dimethyl-1^5,1^6,1^7,1^8,3^1,3^2,3^3,3^4-octahydro-2,6-dioxa-1(4,5)-[1,3]dioxolo[4,5-g]isoquinolina-3(7,1)-isoquinolina-5(1,3),7(1,4)-dibenzenacyclooctaphane]<br><br>CAS: 481-49-2	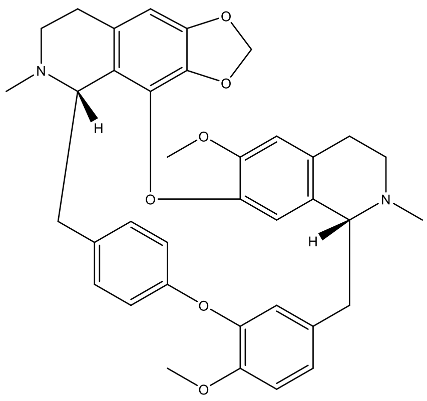	[2]
4	**Quercetin**<br>[2-(3,4-dihydroxyphenyl)-3,5,7-trihydroxy-4*H*-chromen-4-one]<br><br>CAS: 117-39-5	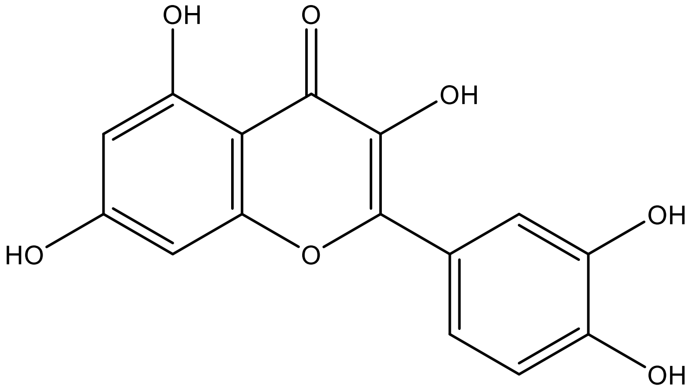	[2, 4, 5]
5	**Catechin**<br>[2-(3,4-dihydroxyphenyl)chromane-3,5,7-triol]<br><br>CAS: 154-23-4	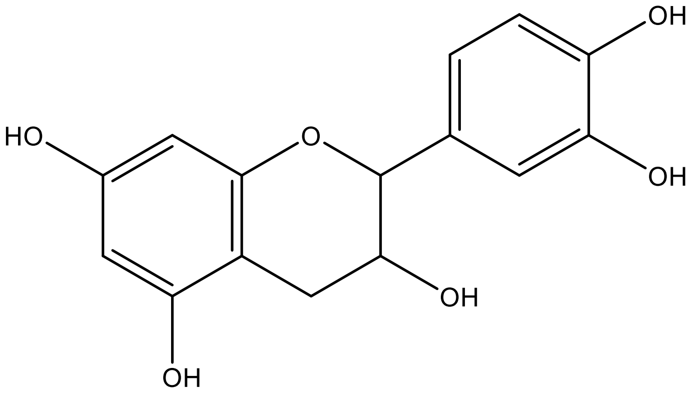	[6]

6	**Ursolic acid**<br>[(1*S*,2*R*,4a*S*,6a*S*,6b*R*,10*S*,12a*R*,12b*R*,14b*S*)-10-hydroxy-1,2,6a,6b,9,9,12a-heptamethyl-1,3,4,5,6,6a,6b,7,8,8a,9,10,11,12,12a,12b,13,14b-octadecahydropicene-4a(2*H*)-carboxylic acid]<br><br>CAS: 77-52-1	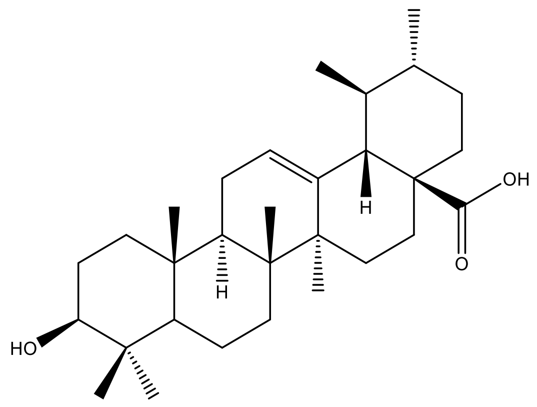	[6]
7	**Bisdemethoxycurcumin**<br>[(1*E*,6*E*)-1,7-bis(4-hydroxyphenyl)hepta-1,6-diene-3,5-dione]<br><br>CAS: 33171-05-0	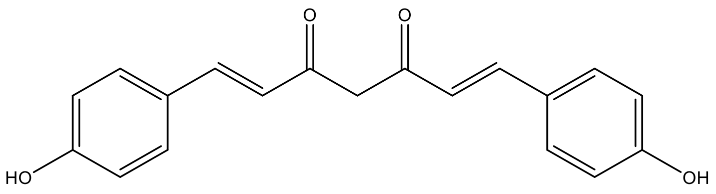	[7]
8	**Curcimun**<br>[(1*E*,6*E*)-1,7-bis(4-hydroxy-3-methoxyphenyl)hepta-1,6-diene-3,5-dione]<br><br>CAS: 458-37-7	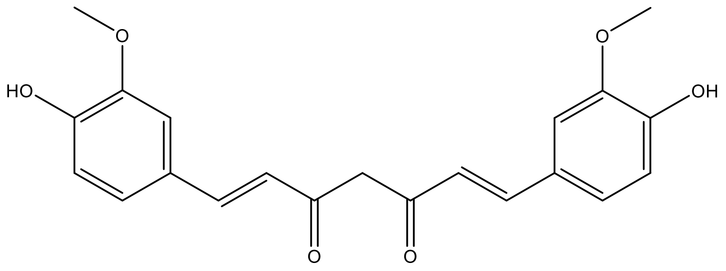	[7]

9	**Demethoxycurcumin**<br>[(1*E*,6*E*)-1-(4-hydroxy-3-methoxyphenyl)-7-(4-hydroxyphenyl)hepta-1,6-diene-3,5-dione]<br><br>CAS: 22608-11-3	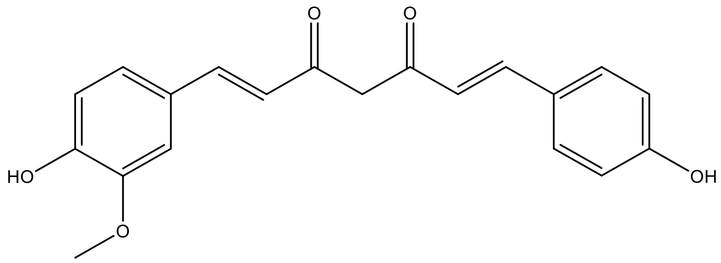	[7]
10	**Galuteolin**<br>[2-(3,4-dihydroxyphenyl)-7-hydroxy-5-(((2*S*,3*R*,4*S*,5*S*,6*R*)-3,4,5-trihydroxy-6-(hydroxymethyl)tetrahydro-2*H*-pyran-2-yl)oxy)-4*H*-chromen-4-one]<br><br>CAS: 5373-11-5	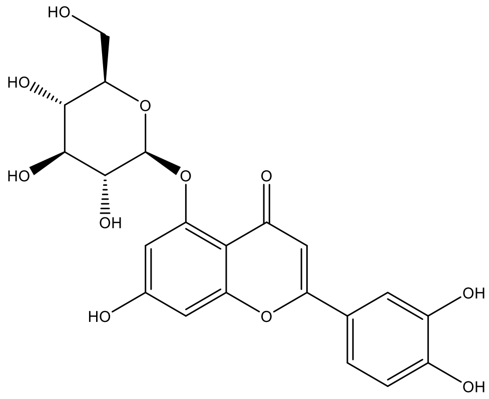	[8]
11	**Emetine**<br>[(2*S*,3*R*,11b*S*)-2-(((*R*)-6,7-dimethoxy-1,2,3,4-tetrahydroisoquinolin-1-yl)methyl)-3-ethyl-9,10-dimethoxy-1,3,4,6,7,11b-hexahydro-2*H*-pyrido[2,1-a]isoquinoline]<br><br>CAS: 483-18-1	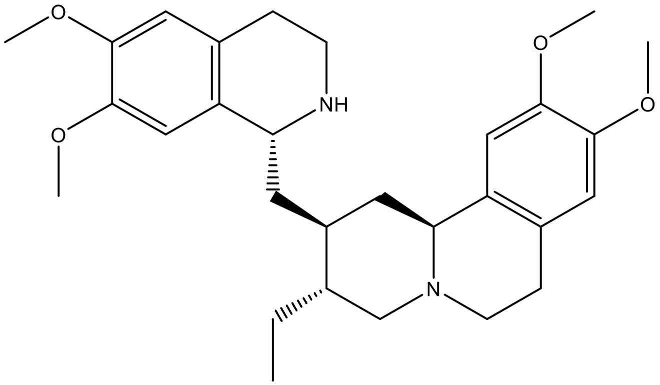	[9]

12	**Andrographolide**<br>[(*S*,*E*)-4-hydroxy-3-(2-((1*R*,4a*S*,5*R*,6*R*,8a*S*)-6-hydroxy-5-(hydroxymethyl)-5,8a-dimethyl-2-methylenedecahydronaphthalen-1-yl)ethylidene)dihydrofuran-2(3*H*)-one]<br><br>CAS: 5508-58-7	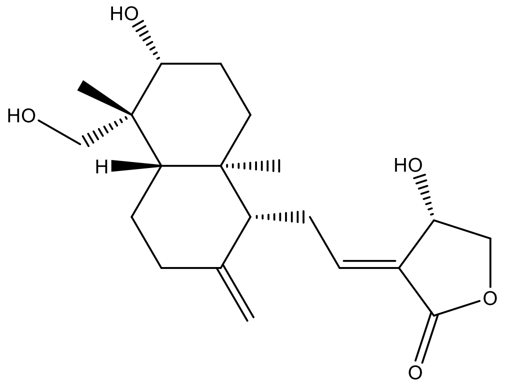	[4]
13	**Kanamycin**<br>[(2*R*,3*S*,4*S*,5*R*,6*R*)-2-(aminomethyl)-6-(((1*R*,2*R*,3*S*,4*R*,6*S*)-4,6-diamino-3-(((2*S*,3*R*,4*S*,5*S*,6*R*)-4-amino-3,5-dihydroxy-6-(hydroxymethyl)tetrahydro-2*H*-pyran-2-yl)oxy)-2-hydroxycyclohexyl)oxy)tetrahydro-2*H*-pyran-3,4,5-triol]<br><br>CAS: 59-01-8	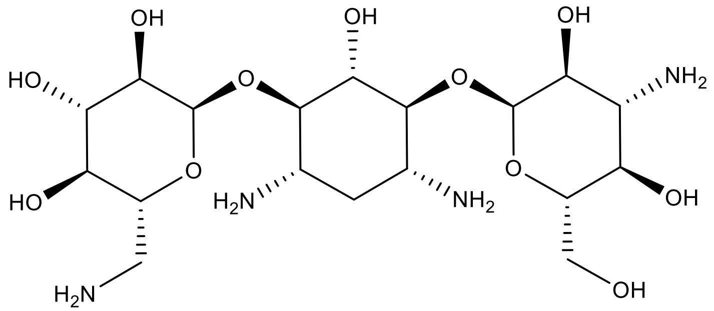	[10]
14	**Tobramycin**<br>[(2*S*,3*R*,4*R*,5*S*,6*R*)-4-amino-2-(((1*S*,2*S*,3*R*,4*S*,6*R*)-4,6-diamino-3-(((2*R*,3*R*,5*S*,6*R*)-3-amino-6-(aminomethyl)-5-hydroxytetrahydro-2*H*-pyran-2-yl)oxy)-2-hydroxycyclohexyl)oxy)-6-(hydroxymethyl)tetrahydro-2*H*-pyran-3,5-diol]<br><br>CAS: 32986-56-4	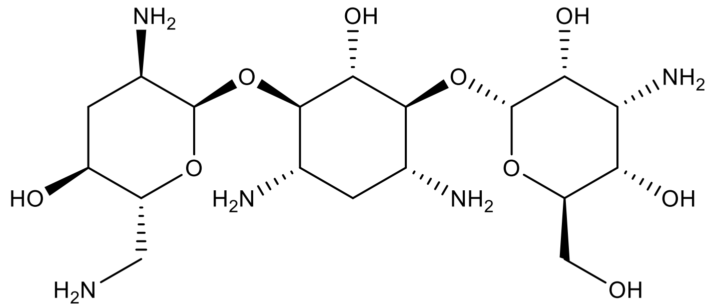	[10]

15	**Hydroxychloroquine**<br>[2-((4-((7-chloroquinolin-4-yl)amino)pentyl)(ethyl)amino)ethan-1-ol]<br><br>CAS: 118-42-3	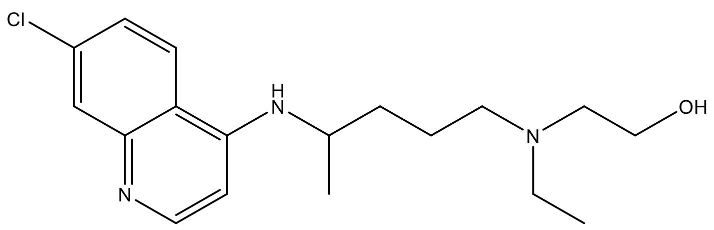	[10, 11]
16	**Remdesivir**<br>[2-ethylbutyl ((((2*R*,3*S*,4*R*,5*R*)-5-(4-aminopyrrolo[2,1-*f*][1,2,4]triazin-7-yl)-5-cyano-3,4-dihydroxytetrahydrofuran-2-yl)methoxy)(phenoxy)phosphoryl)-*L*-alaninate]<br><br>CAS: 1809249-37-3	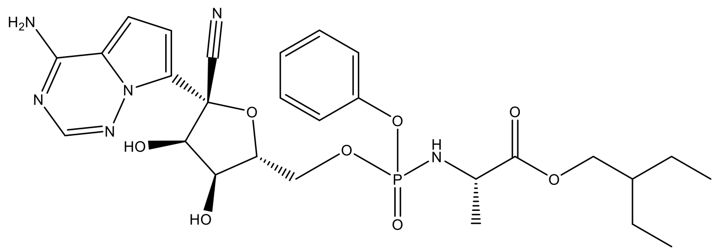	[10]
17	**Dolutegravir**<br>[(4*R*,12a*S*)-*N*-(2,4-difluorobenzyl)-7-hydroxy-4-methyl-6,8-dioxo-3,4,6,8,12,12a-hexahydro-2*H*-pyrido[1',2':4,5]pyrazino[2,1-*b*][1,3]oxazine-9-carboxamide]<br><br>CAS: 1051375-16-6	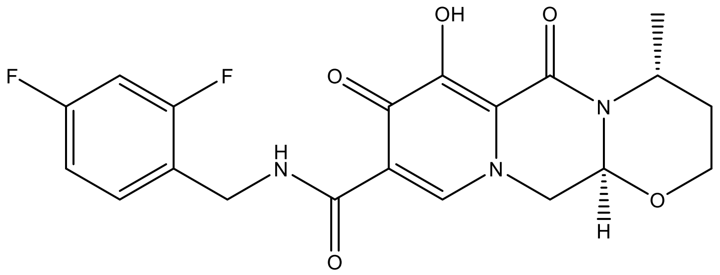	[12]

18	**Lopinavir**<br>[(*S*)-*N*-((2*S*,4*S*,5*S*)-5-(2-(2,6-dimethylphenoxy)acetamido)-4-hydroxy-1,6-diphenylhexan-2-yl)-3-methyl-2-(2-oxotetrahydropyrimidin-1(2*H*)-yl)butanamide]<br><br>CAS: 192725-17-0	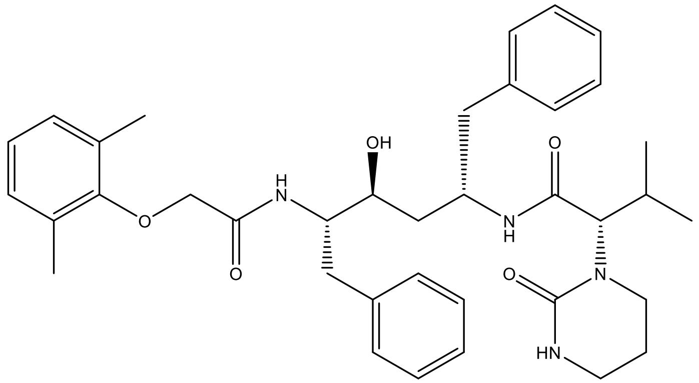	[13]
19	**Ritonavir**<br>[thiazol-5-ylmethyl ((2*S*,3*S*,5*S*)-3-hydroxy-5-((*S*)-2-(3-((2-isopropylthiazol-4-yl)methyl)-3-methylureido)-3-methylbutanamido)-1,6-diphenylhexan-2-yl)carbamate]<br><br>CAS: 155213-67-5	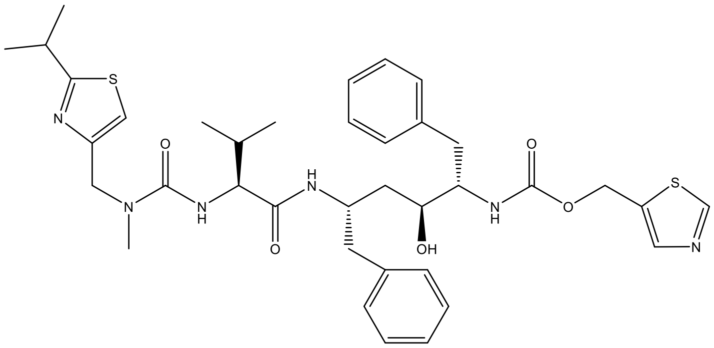	[13]
20	**Oleuropein**<br>[methyl (2*S*,4*S*,*E*)-4-(2-(3,4-dihydroxyphenethoxy)-2-oxoethyl)-3-ethylidene-2-(((3*R*,4*S*,5*S*,6*R*)-3,4,5-trihydroxy-6-(hydroxymethyl)tetrahydro-2*H*-pyran-2-yl)oxy)-3,4-dihydro-2*H*-pyran-5-carboxylate]<br><br>CAS: 32619-42-4	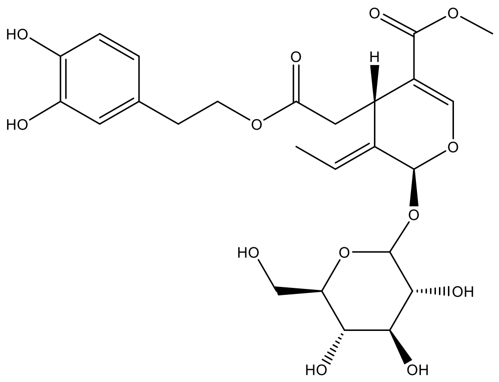	[13]

21	**Cyanidin-3-O-glucoside**<br>[2-(3,4-dihydroxyphenyl)-5,7-dihydroxy-3-(((3*R*,4*S*,5*S*,6*R*)-3,4,5-trihydroxy-6-(hydroxymethyl)tetrahydro-2*H*-pyran-2-yl)oxy)chromenylium]<br><br>CAS: 7084-24-4	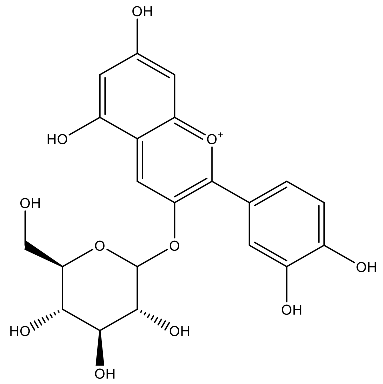	[13]
22	**Epigallocatechin gallate**<br>[(2*R*,3*R*)-5,7-dihydroxy-2-(3,4,5-trihydroxyphenyl)chroman-3-yl 3,4,5-trihydroxybenzoate]<br><br>CAS: 989-51-5	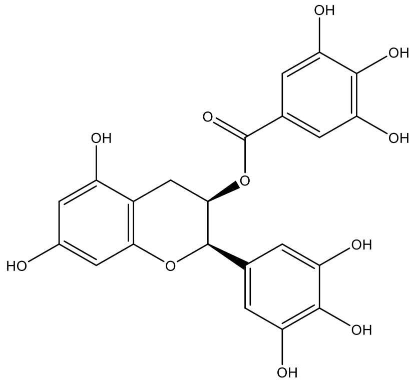	[13]
23	**Neohesperidin**<br>[(*S*)-7-(((2*S*,3*R*,4*S*,5*S*,6*R*)-4,5-dihydroxy-6-(hydroxymethyl)-3-(((2*S*,3*R*,4*R*,5*R*,6*S*)-3,4,5-trihydroxy-6-methyltetrahydro-2*H*-pyran-2-yl)oxy)tetrahydro-2*H*-pyran-2-yl)oxy)-5-hydroxy-2-(3-hydroxy-4-methoxyphenyl)chroman-4-one]<br><br>CAS: 13241-33-3	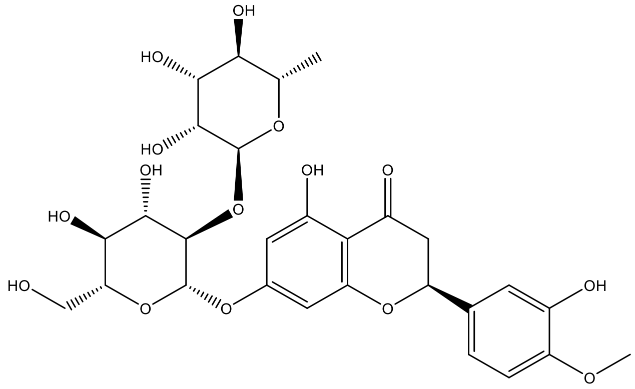	[5]

24	**Riboflavin**<br>[7,8-dimethyl-10-((2*R*,3*R*,4*S*)-2,3,4,5-tetrahydroxypentyl)benzo[*g*]pteridine-2,4(3*H*,10*H*)-dione]<br><br>CAS: 83-88-5	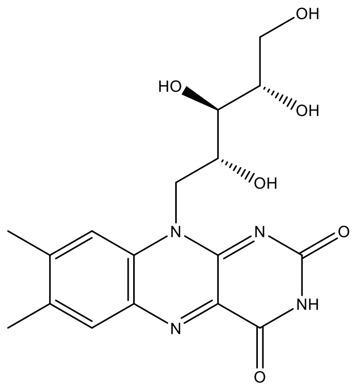	[5]
25	**Telbivudine**<br>[1-((2*S*,4*R*,5*S*)-4-hydroxy-5-(hydroxymethyl)tetrahydrofuran-2-yl)-5-methylpyrimidine-2,4(1*H*,3*H*)-dione]<br><br>CAS: 3424-98-4	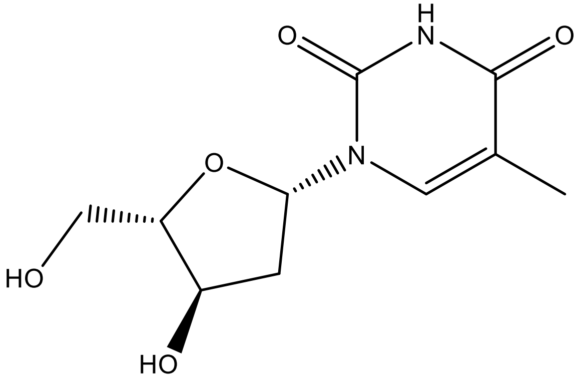	[14]
26	**Oxytetracycline dihydrate**<br>[(4*S*,4a*R*,5*S*,5a*R*,6*S*,12a*R*)-4-(dimethylamino)-1,5,6,10,11,12a-hexahydroxy-6-methyl-3,12-dioxo-3,4,4a,5,5a,6,12,12a-octahydrotetracene-2-carboxamide dihydrate]<br><br>CAS: 6153-64-6	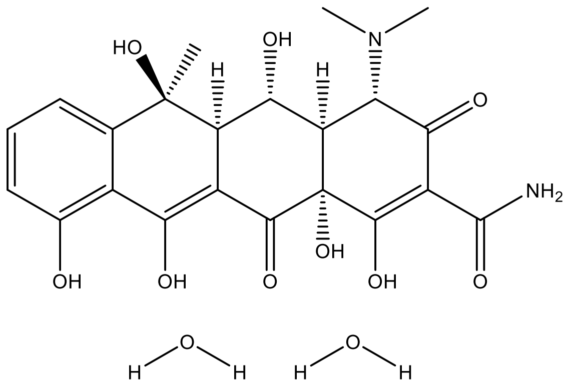	[14]

27	**Methylgallate**<br>[methyl 3,4,5-trihydroxybenzoate]<br><br>CAS: 99-24-1	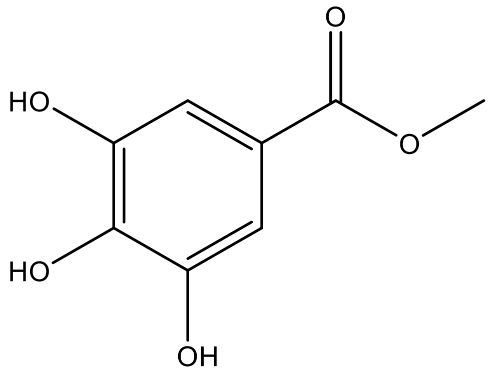 	[14]
28	**2-deoxyglucose**<br>[(3*R*,4*S*,5*R*)-3,4,5,6-tetrahydroxyhexanal]<br><br>CAS: 154-17-6	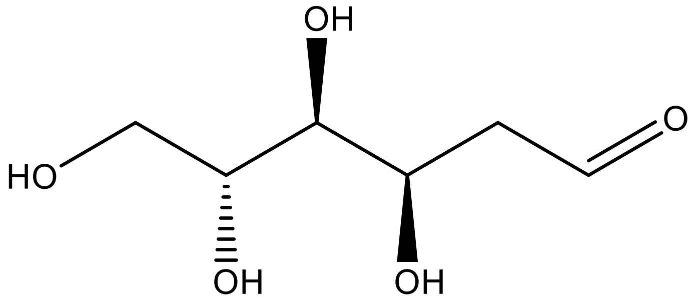 	[14]
29	**Daphnetin**<br>[7,8-dihydroxy-2*H*-chromen-2-one]<br><br>CAS: 486-35-1	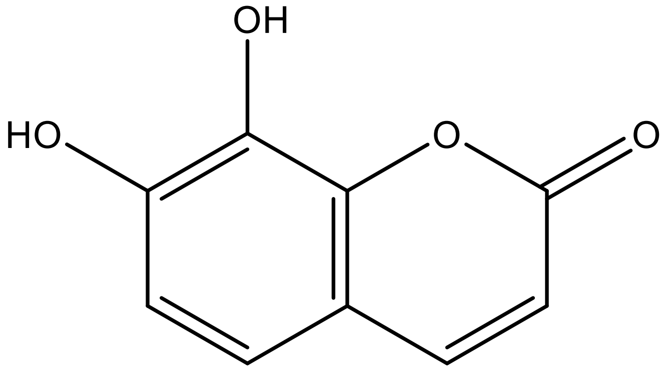 	[14]

30	**Silmitasertib**<br>[5-((3-chlorophenyl)amino)benzo[*c*][2,6]naphthyridine-8-carboxylic acid]<br><br>CAS: 1009820-21-6	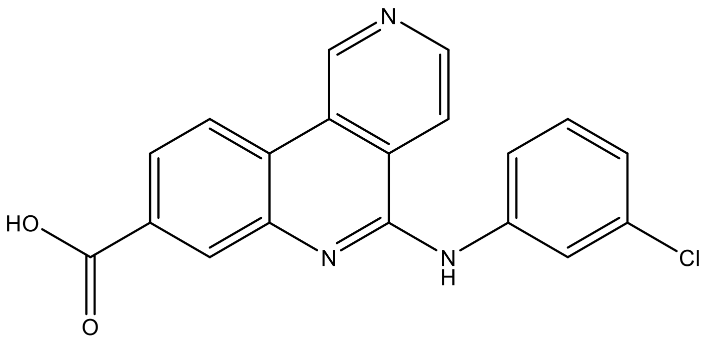	[15]
31[16]	**Chloroquine**<br>[N^4-(7-chloroquinolin-4-yl)-N^1,N^1-diethylpentane-1,4-diamine]<br><br>CAS: 54-05-7	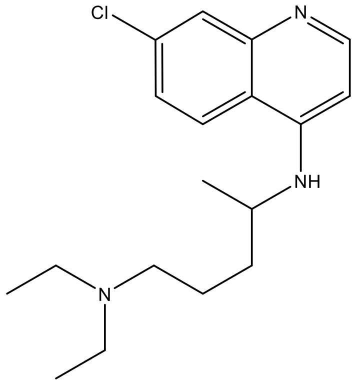	[11]
32	**Sonidegib**<br>[*N*-(6-((2*R*,6*S*)-2,6-dimethylmorpholino)pyridin-3-yl)-2-methyl-4'-(trifluoromethoxy)-[1,1'-biphenyl]-3-carboxamide]<br><br>CAS: 956697-53-3	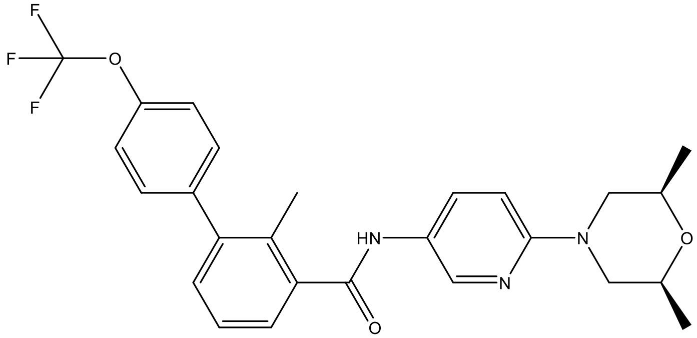	[16]

33	**Bemcentinib**<br>[(*S*)-1-(6,7-dihydro-5*H*-benzo[6,7]cyclohepta[1,2-*c*]pyridazin-3-yl)-*N*^{3}-(7-(pyrrolidin-1-yl)-6,7,8,9-tetrahydro-5*H*-benzo[7]annulen-2-yl)-1*H*-1,2,4-triazole-3,5-diamine]<br><br>CAS: 1037624-75-1	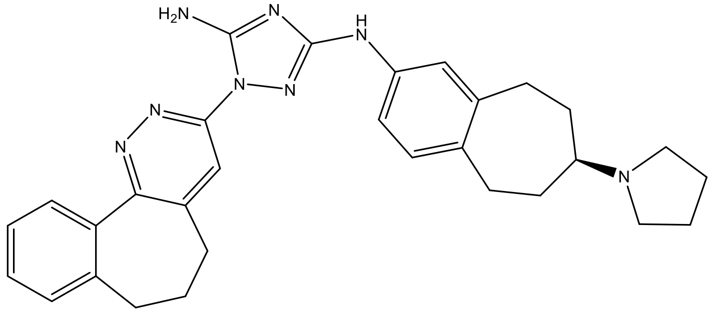	[16]
34	**Itacitinib**<br>[2-(3-(4-(7*H*-pyrrolo[2,3-*d*]pyrimidin-4-yl)-1*H*-pyrazol-1-yl)-1-(1-(3-fluoro-2-(trifluoromethyl)isonicotinoyl)piperidin-4-yl)azetidin-3-yl)acetonitrile]<br><br>CAS: 1334298-90-6	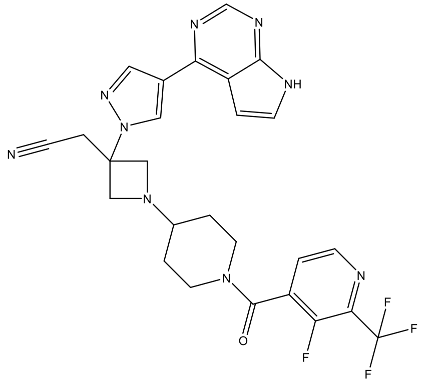	[16]
35	**Phthalocyanine**<br><br>CAS: 574-93-6	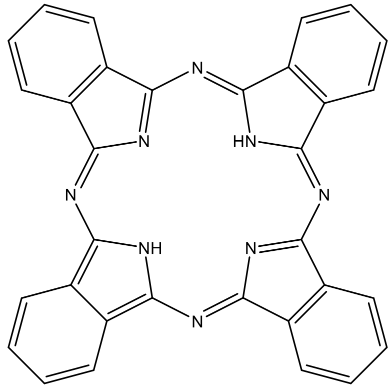	[16]

36	**Acaciin**<br>[5-hydroxy-2-(4-methoxyphenyl)-7-(((2*S*,3*R*,4*S*,5*S*,6*R*)-3,4,5-trihydroxy-6-((((2*R*,3*R*,4*R*,5*R*,6*S*)-3,4,5-trihydroxy-6-methyltetrahydro-2*H*-pyran-2-yl)oxy)methyl)tetrahydro-2*H*-pyran-2-yl)oxy)-4*H*-chromen-4-one]<br><br>CAS: 480-36-4	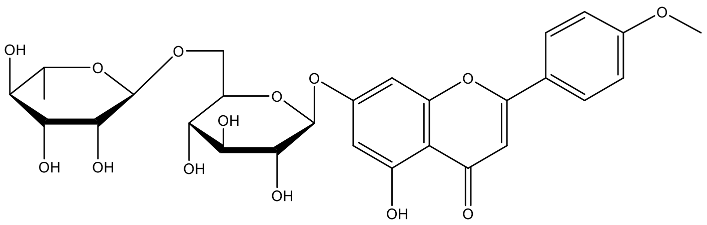	[17]
37	**Didymin**<br>[5-hydroxy-2-(4-methoxyphenyl)-7-(((2*S*,3*R*,4*S*,5*S*,6*R*)-3,4,5-trihydroxy-6-((((2*R*,3*R*,4*R*,5*R*,6*S*)-3,4,5-trihydroxy-6-methyltetrahydro-2*H*-pyran-2-yl)oxy)methyl)tetrahydro-2*H*-pyran-2-yl)oxy)chroman-4-one]<br><br>CAS: 14259-47-3	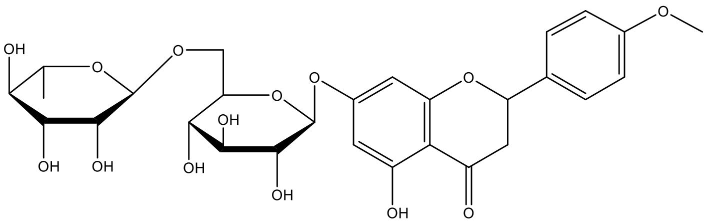	[17]

References

1. Singh, R., et al., *In-silico evaluation of bioactive compounds from tea as potential SARS-CoV-2 nonstructural protein 16 inhibitors.* J Tradit Complement Med, 2022. **12**(1): p. 35-43.
2. Hossain, R., et al., *In Silico Screening of Natural Products as Potential Inhibitors of SARS-CoV-2 Using Molecular Docking Simulation.* Chin J Integr Med, 2022. **28**(3): p. 249-256.
3. El-Hawary, S.S., et al., *Metabolomic profiling of three Araucaria species, and their possible potential role against COVID-19.* J Biomol Struct Dyn, 2022. **40**(14): p. 6426-6438.
4. Saliu, T.P., et al., *Molecular docking and pharmacokinetic studies of phytocompounds from Nigerian Medicinal Plants as promising inhibitory agents against SARS-CoV-2 methyltransferase (nsp16).* J Genet Eng Biotechnol, 2021. **19**(1): p. 172.
5. Al-Sanea, M.M., et al., *Strawberry and Ginger Silver Nanoparticles as Potential Inhibitors for SARS-CoV-2 Assisted by In Silico Modeling and Metabolic Profiling.* Antibiotics (Basel), 2021. **10**(7).
6. Bhardwaj, A., S. Sharma, and S.K. Singh, *Molecular Docking Studies to Identify Promising Natural Inhibitors Targeting SARS-CoV-2 Nsp10-Nsp16 Protein Complex.* Turk J Pharm Sci, 2022. **19**(1): p. 93-100.
7. Alici, H., H. Tahtaci, and K. Demir, *Design and various in silico studies of the novel curcumin derivatives as potential candidates against COVID-19 -associated main enzymes.* Comput Biol Chem, 2022. **98**: p. 107657.
8. Vijayan, V., et al., *Identification of promising drug candidates against NSP16 of SARS-CoV-2 through computational drug repurposing study.* J Biomol Struct Dyn, 2021. **39**(17): p. 6713-6727.
9. Snoussi, M., et al., *Emetine, a potent alkaloid for the treatment of SARS-CoV-2 targeting papain-like protease and non-structural proteins: pharmacokinetics, molecular docking and dynamic studies.* J Biomol Struct Dyn, 2022. **40**(20): p. 10122-10135.
10. Rampogu, S. and K.W. Lee, *Pharmacophore Modelling-Based Drug Repurposing Approaches for SARS-CoV-2 Therapeutics.* Front Chem, 2021. **9**: p. 636362.
11. Gentile, D., et al., *New Anti SARS-Cov-2 Targets for Quinoline Derivatives Chloroquine and Hydroxychloroquine.* Int J Mol Sci, 2020. **21**(16).
12. Malik, A., et al., *In silico screening of phytochemical compounds and FDA drugs as potential inhibitors for NSP16/10 5' methyl transferase activity.* J Biomol Struct Dyn, 2023. **41**(1): p. 221-233.
13. Liang, J., et al., *In silico investigation of potential small molecule inhibitors of the SARS-CoV-2 nsp10-nsp16 methyltransferase complex.* Chem Phys Lett, 2021. **774**: p. 138618.
14. Maurya, S.K., et al., *Virtual screening, ADME/T, and binding free energy analysis of anti-viral, anti-protease, and anti-infectious compounds against NSP10/NSP16 methyltransferase and main protease of SARS CoV-2.* J Recept Signal Transduct Res, 2020. **40**(6): p. 605-612.
15. Gorgulla, C., et al., *A multi-pronged approach targeting SARS-CoV-2 proteins using ultra-large virtual screening.* iScience, 2021. **24**(2): p. 102021.
16. Encinar, J.A. and J.A. Menendez, *Potential Drugs Targeting Early Innate Immune Evasion of SARS-Coronavirus 2 via 2'-O-Methylation of Viral RNA.* Viruses, 2020. **12**(5).
17. Albohy, A., et al., *Multitarget in silico studies of Ocimum menthiifolium, family Lamiaceae against SARS-CoV-2 supported by molecular dynamics simulation.* J Biomol Struct Dyn, 2022. **40**(9): p. 4062-4072.
