# Supplementary material for: Assay development and screening of inhibitors targeting the SARS-CoV-2 2′-O-methyltransferase NSP16
Source: Pharm Sci Adv. 2025 May 21;3:100076. doi: 10.1016/j.pscia.2025.100076 (PMC12709967; doi:10.1016/j.pscia.2025.100076)
Supplement: Multimedia component 2 [file mmc2.pdf]

Compound	Structure	EC_{50} curve
MC87	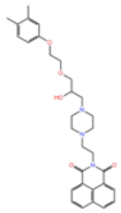	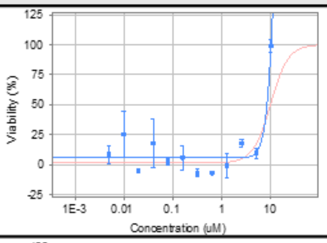
MC100	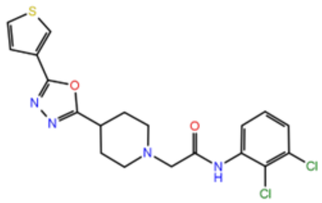	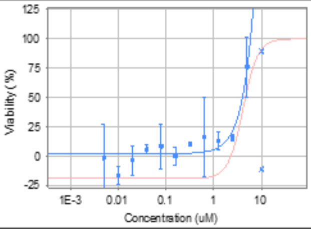
MC76	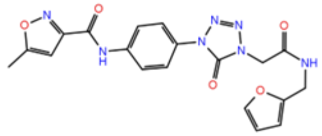	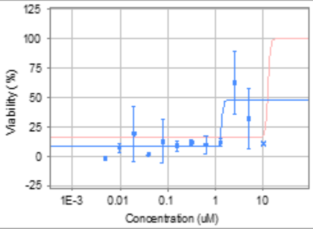
MC77	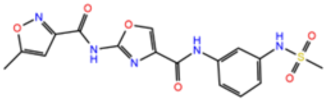	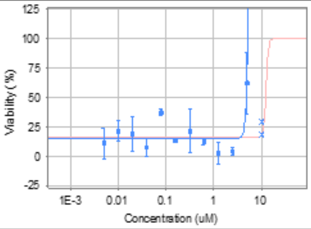
MC64	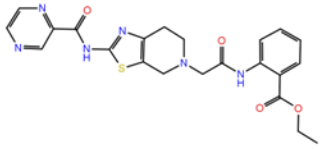	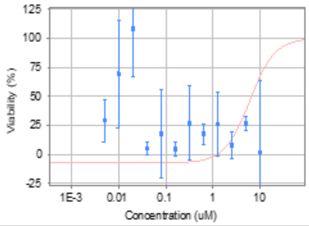
MC119	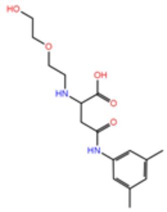	

Supplementary Figure S1. Cellular assay of top hits. EC_{50} curve and structure of top hits selected by the biochemical assay.

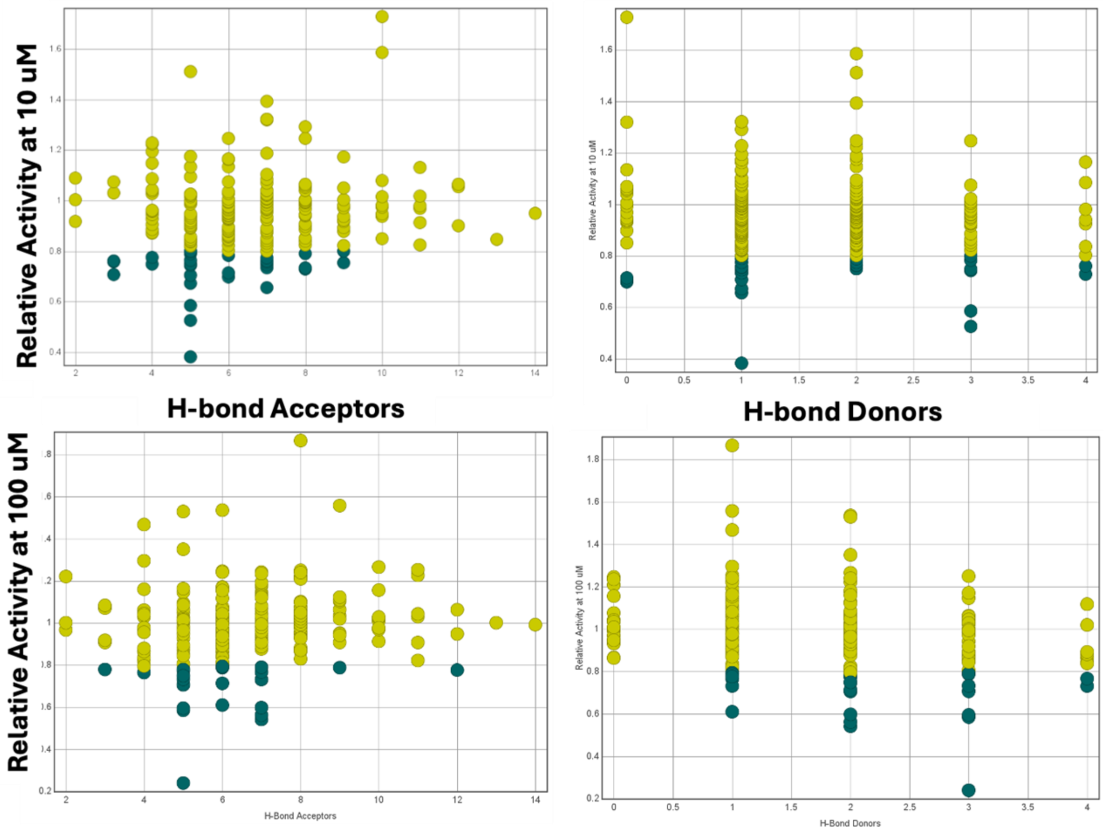

Supplementary Figure S2. Hydrogen Bond donor & acceptor trends. Optimal H-bond acceptors 5, optimal donors 3 using 10 μM and 100 μM experimental data. The data was visualized using Data Warrior.

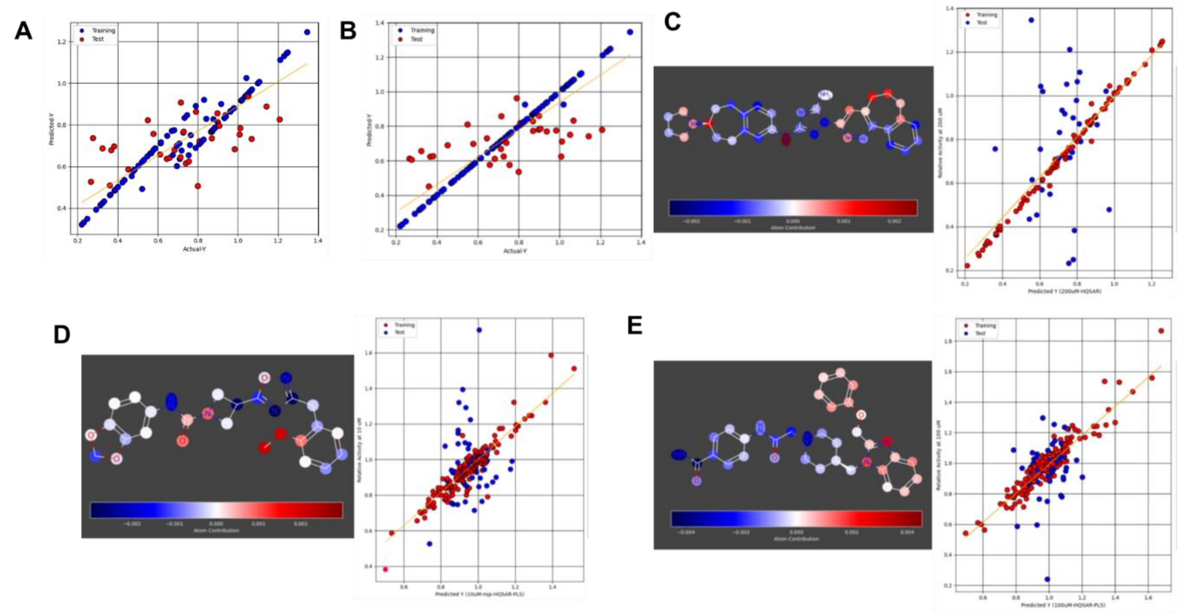

Supplementary Figure S3. 2D-QSAR and Auto-QSAR model generated from experimental data. (A) Auto-QSAR model generated from 200uM experimental data using Support Vector Regression (SVR). (B) Auto-QSAR model generated from 200uM experimental data using k-nearest neighbor (kNN). (C) Using AutoQSAR on 200 µM data us PLS. (D) Using AutoQSAR on 100 µM data us PLS. (E) Using AutoQSAR on 10 µM data us PLS.

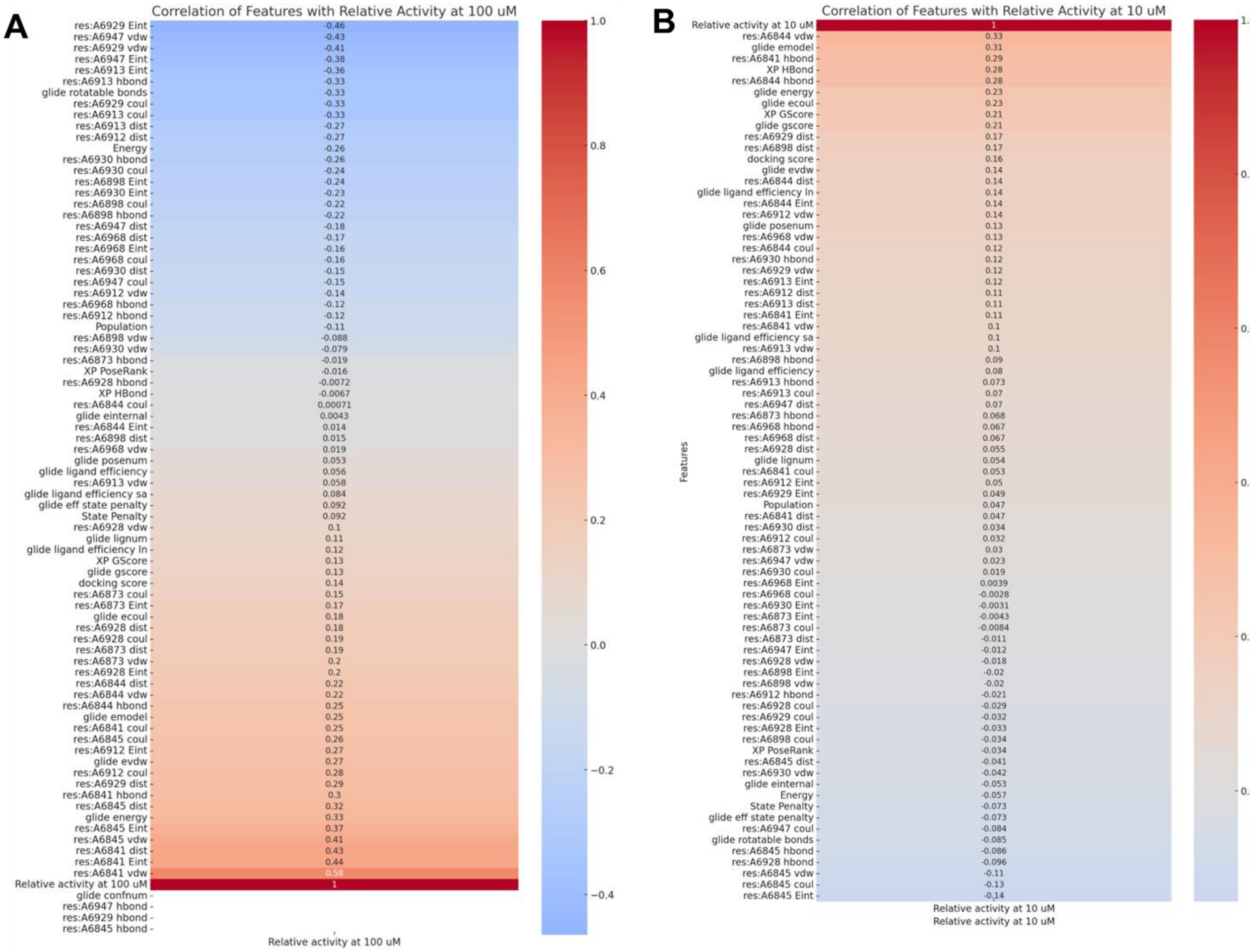

Supplementary Figure S4. Correlation of Per-residue scoring with relative activity at (A) 100 μM and (B) 10 μM.

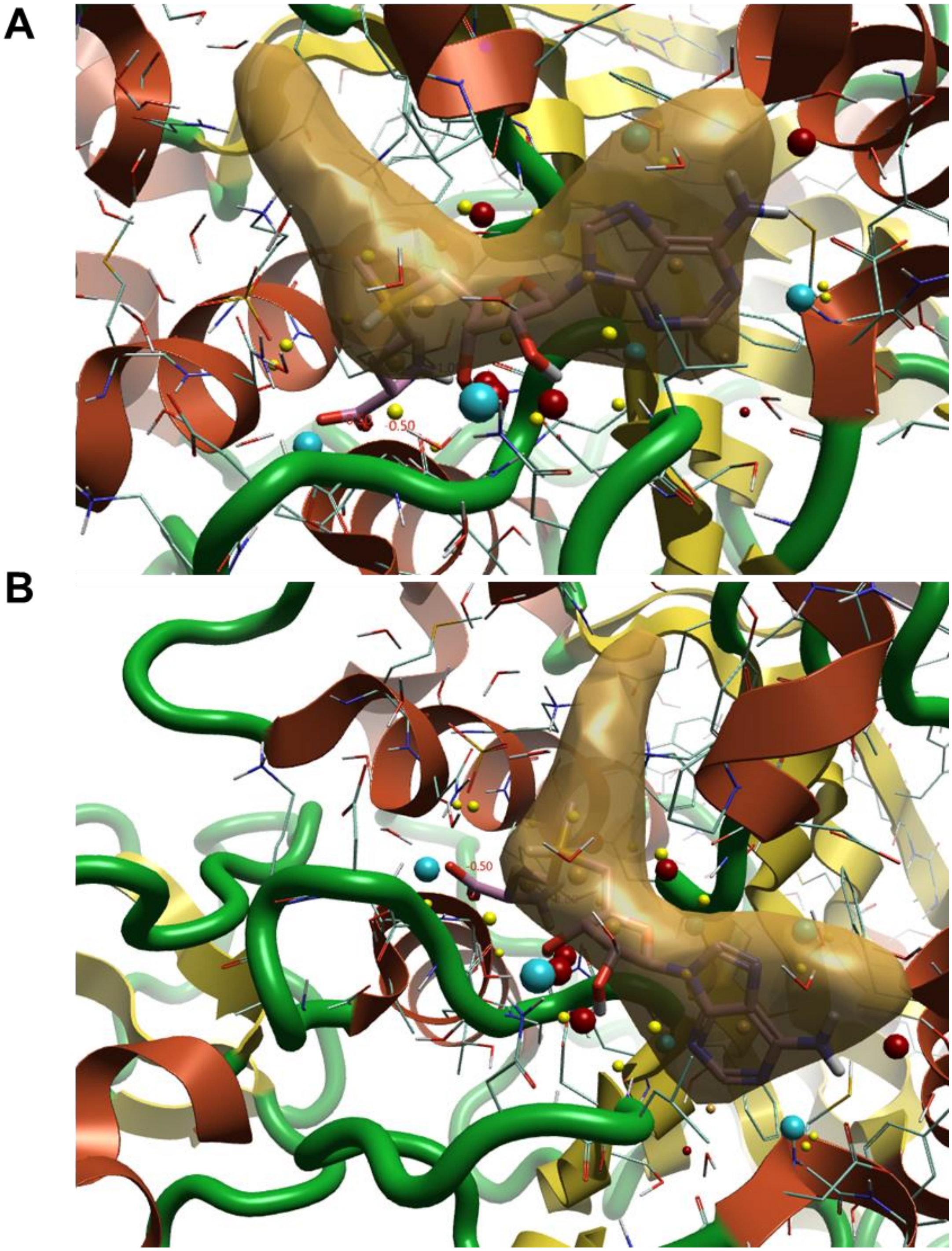

Supplementary Figure S5: Average hydrophobic contributions correlated with potent compounds using Cresset Activity Atlas using (A) 10 μM experimental data and (B) 100 μM experimental data.

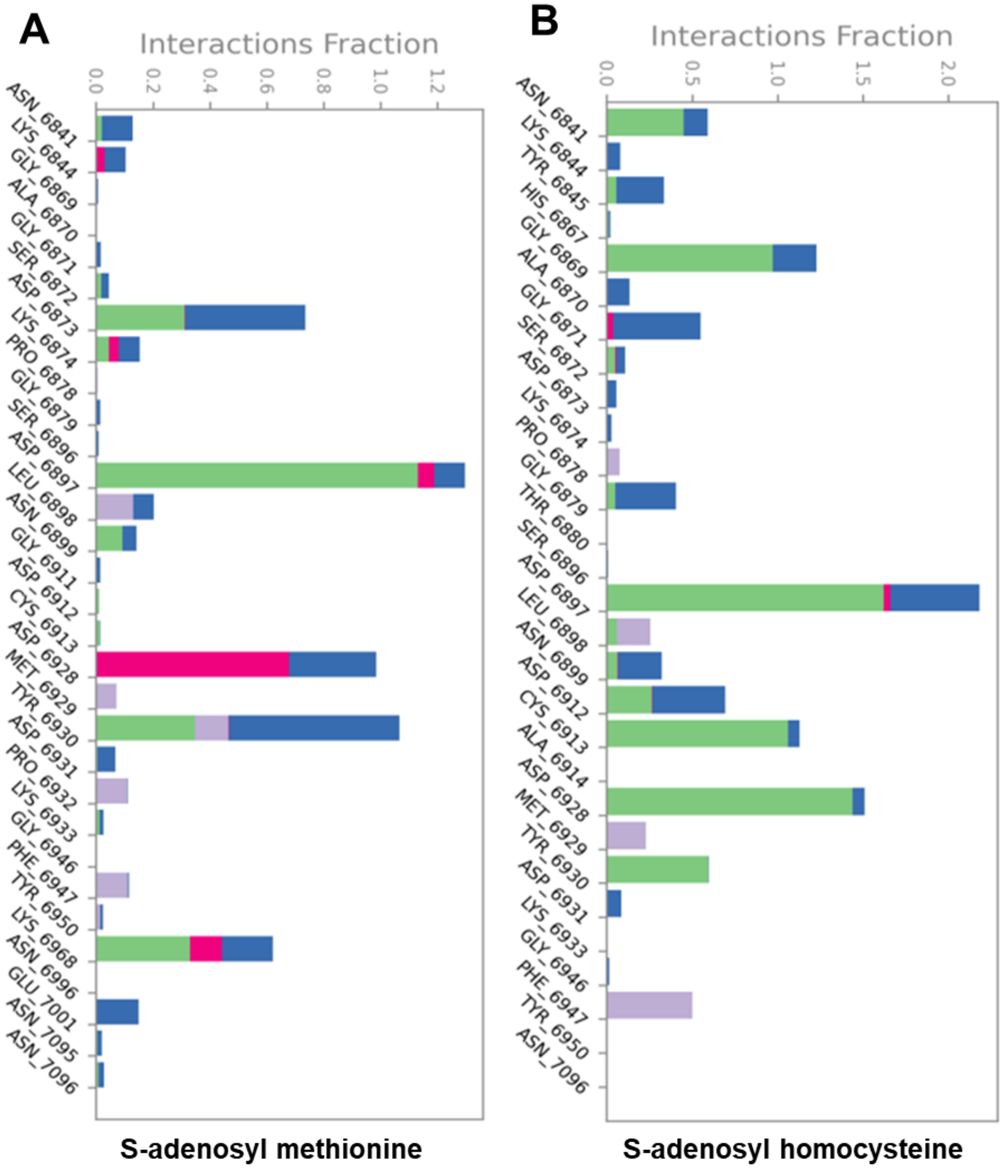

Supplementary Figure S6: Comparison of predicted interaction from molecular dynamic simulations of S-adenosyl-methionine (SAM) and S-adenosyl-homocysteine (SAH) bound to NSP16. (A) SAM: Key ionic interactions Asp 6928 and Lys 6968, H-bond/Water bridges at Tyr 6930 and Asp 6897. (B) SAH: Similar interacting pattern at Asp 6897, no ionic interaction Lys 6968. Gain H-bonding/water bridging interaction Asp 6912, 6928

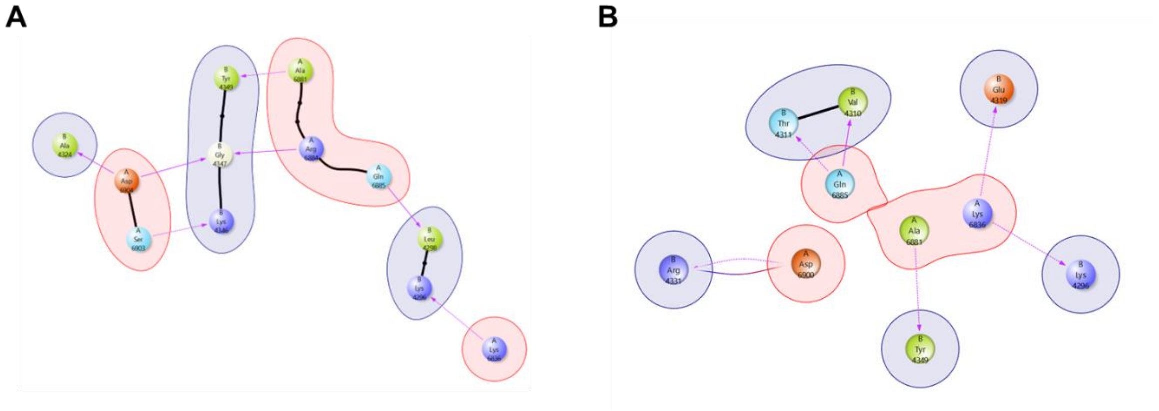

Supplementary Figure S7: Protein: Protein interaction analysis comparing SAM-bound NSP16 and Bemcentinib-bound NSP10. (A) Protein Protein interactions of SAM-bound NSP16/NSP10 interface. (B) Protein: Protein interactions of Bemcentinib-bound NSP10/NSP16 interface.
