# Supplementary figures and images for: Assay development and screening of inhibitors targeting the SARS-CoV-2 2′-O-methyltransferase NSP16
Source: Pharm Sci Adv. 2025 May 21;3:100076. doi: 10.1016/j.pscia.2025.100076 (PMC12709967; doi:10.1016/j.pscia.2025.100076)

## Slide 1
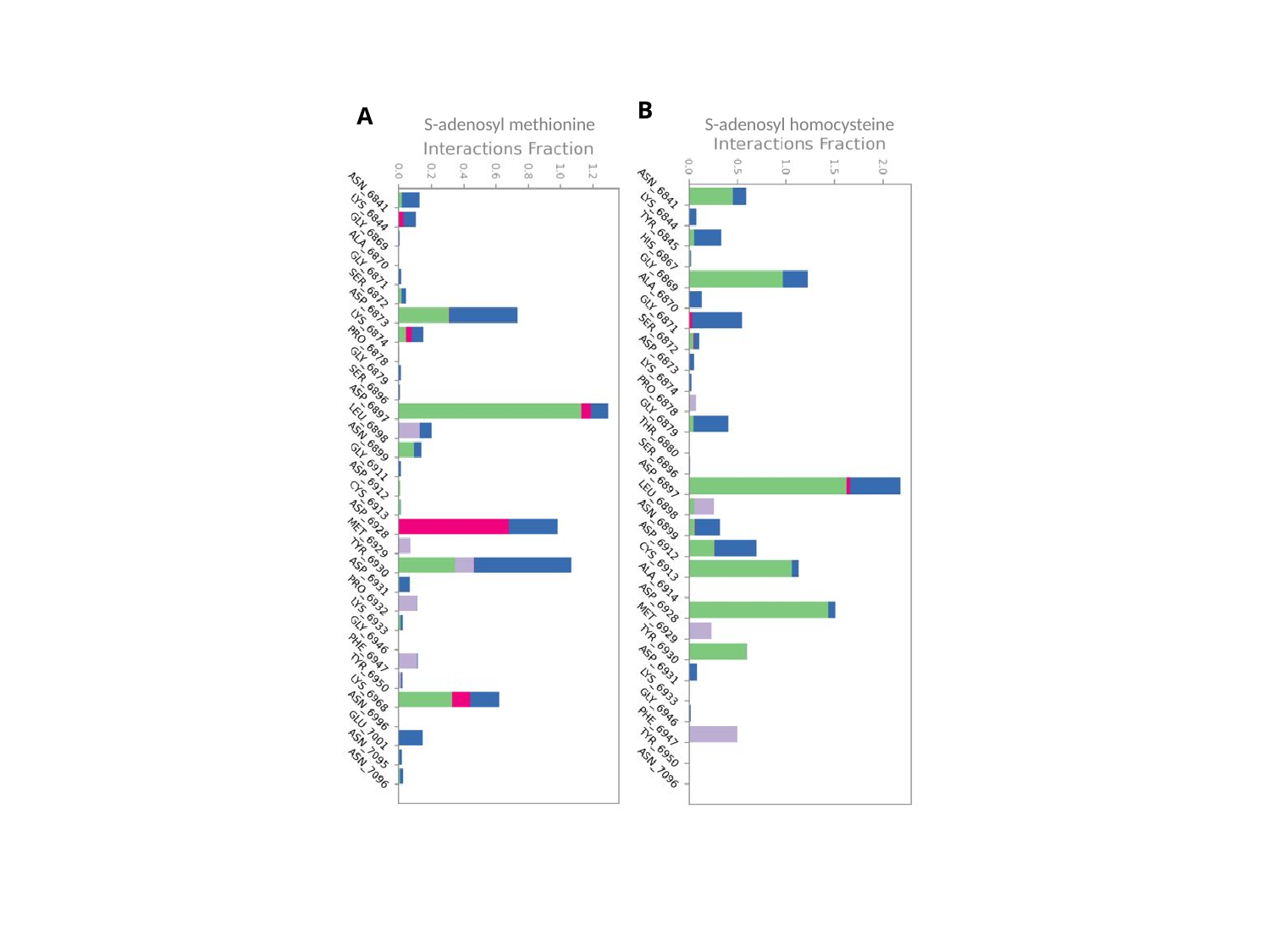

B
A
S-adenosyl methionine S-adenosyl homocysteine

Supplement: Multimedia component 3 [file mmc3.pptx]
